# Morphea, Eosinophilic Fasciitis and Cancer: A Scoping Review

**DOI:** 10.3390/cancers15184450

**Published:** 2023-09-07

**Authors:** Maxine Joly-Chevrier, Alexa Gélinas, Stephanie Ghazal, Sarah Moussa, Catherine C. McCuaig, Maryam Piram, Alexandra Mereniuk, Ivan V. Litvinov, Mohammed Osman, Kevin Pehr, Elena Netchiporouk

**Affiliations:** 1 Faculty of Medicine, Université de Montréal, Montreal, QC H3T 1J4, Canada; 2 Division of Dermatology, McGill University Health Centre, Montreal, QC H4A 3J1, Canada; 3 Faculty of Medicine, McGill University, Montreal, QC H3G 2M1, Canada; 4 Division of Pediatric Dermatology, Sainte-Justine Hospital, Montreal, QC H3T 1C5, Canada; 5 Division of Dermatology, Sacre Coeur Hospital, Montreal, QC H4J 1C5, Canada; 6 Division of Rheumatology, University of Alberta, Edmonton, AB T6G 2G3, Canada; 7 Division of Dermatology, Jewish General Hospital, McGill University, Montreal, QC H3N 1V4, Canada

**Keywords:** localized scleroderma, morphea, eosinophilic fasciitis, cancer, malignancy, paraneoplastic, radiation induced scleroderma, chemotherapy, immune checkpoint inhibitors, immunotherapy

## Abstract

**Simple Summary:**

Morphea and eosinophilic fasciitis (EF) are cutaneous autoimmune fibrosing diseases. We conducted a scoping review following PRISMA-ScR guidelines to ascertain the association between cancer and morphea/EF, focusing specifically on the paraneoplastic phenomenon, risk of subsequent cancer and development of morphea/EF as a consequence of cancer treatment. We identified that morphea patients, particularly those with generalized disease, might be at an increased risk of secondary malignancy, notably skin and pancreatic cancer. EF, on the other hand, occurred as a paraneoplastic disease in 10% of patients, primarily associated with hematologic malignancies. While reports of radiotherapy and chemotherapy-induced morphea are numerous, immunotherapy-induced morphea/EF cases are emerging. Interestingly, all immunotherapy-induced cases occurred with PD-1 inhibitors.

**Abstract:**

Morphea is an autoimmune fibrotic skin disease. Eosinophilic fasciitis (EF) is considered to belong to the severe spectrum of morphea. We conducted a scoping review assessing the risk of secondary cancer among morphea/EF patients, paraneoplastic morphea/EF and morphea/EF developing secondary to cancer therapy. The search was conducted using MEDLINE, Embase, Cochrane databases for articles published from inception to September 2022 following the Preferred Reporting Items for Systematic reviews and Meta-Analyses for Scoping Reviews (PRISMA-ScR) guidelines with no language or date restrictions. Two hundred and one studies were included. Of these, 32 studies reported on secondary cancer in morphea/EF patients, 45 on paraneoplastic morphea/EF and 125 on cancer-treatment-induced morphea/EF. While the current evidence remains limited, data suggest an increased risk of secondary cutaneous and possibly pancreatic malignancy in morphea patients, particularly the generalized subtype. There were insufficient data for EF. On the other hand, paraneoplastic morphea was anecdotal, whereas several observational studies suggested that ~10% of EF cases may be paraneoplastic, primarily in the context of hematologic malignancies. Radiotherapy-induced morphea is rare, seen in ~0.2% of treated patients and is usually localized to the treatment site, except in patients with pre-existing autoimmunity. While chemotherapy-induced cases are reported, immunotherapy morphea/EF cases are emerging and are preferentially seen with PD-1 and not CTLA-4 inhibitors. This study is limited by the type of articles included (case reports, case series and observational studies), and hence, additional research on this important topic is needed.

## 1. Introduction

Morphea, also referred to as localized scleroderma, is an autoimmune fibrosing skin disease with an estimated lifetime prevalence of 200 per 100,000 and a female predominance (ratio of 3:1) [1,2]. While the pathogenesis remains unknown, morphea is thought to be triggered by external factors (e.g., viral infection, trauma, radiation, and medication) in genetically predisposed individuals [3,4,5]. Morphea is characterized by an initial T-cell-mediated inflammatory phase followed by fibrosis [5,6,7]. Although less prominent than in systemic sclerosis (SSc), vasculopathy changes have also been described [5,6,7]. Over the course of years, disease resolution is expected, however, with residual skin atrophy and/or hyperpigmentation [5,6,7]. Permanent functional and/or cosmetic sequela are not rare [5,6,7]. Eosinophilic fasciitis (EF) is another rare autoimmune fibrotic skin disease that is considered to belong to the severe morphea spectrum [8].

The increased risk of malignancy in patients with systemic fibrosing chronic autoimmune diseases (e.g., SSc) has been previously described [9,10]. While the underlying mechanism behind cancer predisposition in autoimmunity is unclear, multiple factors are likely to contribute, including shared risk factors, chronic inflammation, premature immunosenescence and/or impaired DNA repair/genomic mutations and therapy-related immunosuppression [11,12,13]. Recently, a higher risk of cancer in patients with morphea was suggested [14]. 

On the other hand, immune dysregulation may also present as a paraneoplastic phenomenon, such as that seen in dermatomyositis or paraneoplastic pemphigus [15,16,17]. Cancer neoantigens may drive these inflammatory signals [18]. Previous studies suggested that underlying cancer may be identified in up to 10% of EF patients, and case reports of paraneoplastic morphea were also published [19]. 

Due to limited data, there are currently no recommendations regarding baseline or long-term screening for malignancy in morphea or EF patients. Hence, we sought to conduct a scoping review to explore the evidence to date regarding the risk of (1) secondary malignancy in morphea/EF patients, (2) paraneoplastic morphea/EF, and (3) morphea/EF arising due to cancer therapy.

## 2. Materials and Methods

The Preferred Reporting Items for Systematic Reviews and Meta-Analyses extension for Scoping Reviews (PRISMA-ScR) guidelines were followed. MEDLINE, Embase and Cochrane databases were searched independently by 2 authors (MJC, AG) for original manuscripts published from inception to September 2022. The following keywords and meSH terms were searched: (localized scleroderma OR morphea or eosinophilic fasciitis) AND (carcinom* OR neoplas* OR adenocarcinom* OR cancer* OR tumor* OR tumour* OR sarcom* OR adenom* OR lymphom* OR myeloproliferative or myeloma OR immunotherapy OR immune checkpoint inhibitor OR pembrolizumab OR nivolumab OR ipilimumab OR atezolizumab OR durvalumab OR cemiplimab OR avelumab OR radiotherapy OR radiation therapy OR radiation treatment OR chemotherapy OR chemotherapeutic agent OR antineoplastic agents OR cancer treatment OR cancer therapy). The full search strategy is provided in Supplementary Materials. Inclusion criteria were articles on morphea/EF human patients who were diagnosed with cancer and/or received cancer treatment without restriction to language, study design, sample size or year of publication. Publications reporting on SSc, other conditions (e.g., graft versus host disease) and benign neoplasms were excluded. All types of articles were included except for review articles and conference abstracts. Abstracts and full texts were screened for eligibility by MJC and AG, and in case of disagreement, EN was consulted. Manual searches of references of included manuscripts were performed to reveal additional articles. The articles that were not obtained despite 2 attempts to contact authors and through inter-library loan agreement were excluded. For included articles, the following data were extracted from each study by two independent reviewers (MJC and AG): patient demographics (number of patients, age, sex, race), characteristics of morphea/EF (morphea subtype, time to disease onset), cancer treatment (type of therapy, time to disease onset after cancer therapy initiation) and/or cancer characteristics (cancer type, time to cancer onset). The type of morphea was determined according to the Padua classification [20,21]. Among morphea/EF patients who developed cancer following their diagnosis, a paraneoplastic case was determined if the authors concluded it was a paraneoplastic case and/or when the cancer occurred within 3 years of morphea/EF onset as previously defined [22]. Whereas the occurrence of cancer more than 3 years after morphea/EF diagnosis was included in the risk of cancer in morphea/EF patients’ section. 

## 3. Results

**Figure 1:**
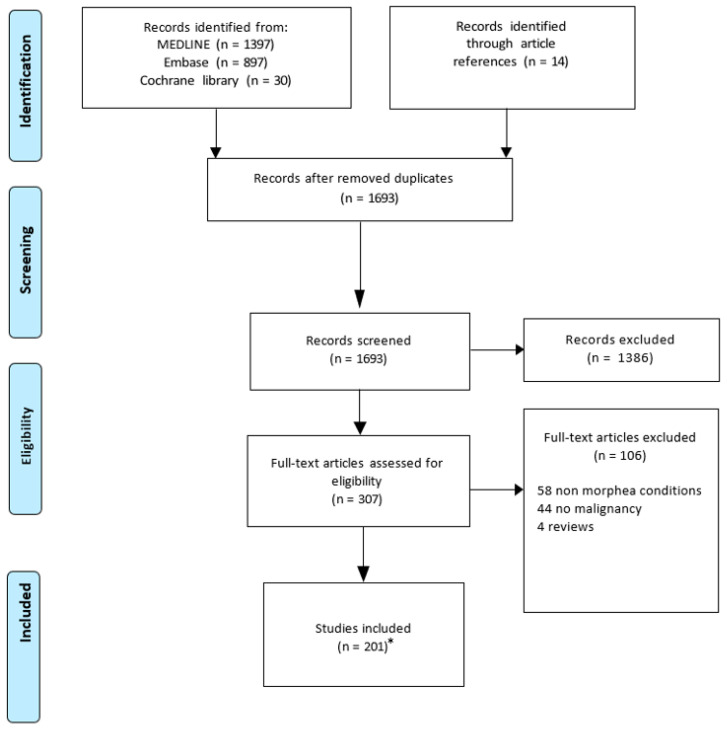
The Preferred Reporting Items of Systematic Reviews and Meta-Analysis (PRISMA) guidelines for Scoping Reviews Search Flow Diagram. * While 201 articles were included, 1 article was discussed in 2 sections.

Of 1683 articles screened, 201 articles including 561 morphea and 79 EF patients were included (published between 1952 and 2022) (Figure 1, Table S1). 

### 3.1. The Risk of Cancer in Morphea Patients 

Twenty-five articles reported a new onset of cancer in morphea patients. Of these, 22 were case reports (22/25 or 88.0%), 2 retrospective case-control studies (2/25 or 8.0%) and 1 was a cross-sectional study (1/25 or 4.0%). In total, 331 morphea patients developed cancer. In 60% of cases (201/331), the diagnosis of morphea was confirmed by histopathology. Morphea subtypes were generalized in 43.5% of patients (10/23), pansclerotic in 26.1% (6/23), circumscribed in 21.7% (5/23) and linear in 8.7% (2/23). Most patients were females (94/112 or 83.9%) and White (88/93 or 94.6%). There were 11 juvenile morphea cases, among which 2 (2/11 or 18.2%) were diagnosed with cancer during adolescence, while others were diagnosed in adulthood. Mean age at cancer diagnosis was 38.8 (Standard Deviation (SD), 18.7 [*n* = 187]) years old. Mean time to cancer onset was 15.0 years (SD, 6.9 [*n* = 21]).

The most common cancer was pancreatic (166/331 or 50.2%), followed by skin cancer (117/331 or 35.3%), gynecologic (vagina, endometrial) (37/331, 11.2%), breast (17/331, 5.1%) and hematological malignancies (2/331, <1.0%). For skin cancer specifically, 61 cases (18.4%) were squamous cell carcinoma (SCC), 30 (9.1%) basal cell carcinoma (BCC) and 16 (4.7%) melanoma. However, this information should be interpreted with caution as the 3 observational studies (detailed below) investigated specifically pancreatic cancer, breast/gynecologic malignancies, and skin neoplasms. No studies focusing on other types of malignancies or malignancy risk in general were identified in our search.

A US population-based retrospective case-control study used insurance databases and physician billing claims (International Classification of Diseases 9th edition (ICD-9)) to investigate the risk of pancreatic cancer in patients with autoimmune diseases, including morphea. The control group was randomly selected in a ratio of 4:1 from a representative sample of Medicare-enrolled beneficiaries without cancer or from the Surveillance Epidemiology and End Results Program (SEER)-Medicare Patient Entitlement Diagnosis Summary File (PEDSF). In total, 166 morphea patients were diagnosed with pancreatic cancer (155 with pancreatic ductal adenocarcinoma). An increased risk of pancreatic cancer with an odds ratio (OR) of 1.27 (95% confidence interval (CI), 1.06–1.52) was seen, particularly for pancreatic ductal adenocarcinoma (OR 1.24; 95% CI, 1.03–1.48) when compared to the control group even after adjusting for multiple confounders (e.g., demographics, life habits and relevant chronic illnesses) [23]. 

A Swedish study similarly searched for incident cases of breast and gynecologic malignancies in a national population-based database among patients diagnosed with 33 autoimmune diseases, including 708 patients diagnosed with morphea based on ICD 7 to 10 codes. No increased risk of breast cancer was seen, whereas a higher-than-expected incidence of other female genital cancers (standardized incidence ratio (SIR) 35.88; 95% CI, 24.01–51.58) was suggested [24]. However, the hazard ratios (HR) in morphea patients compared to controls (without autoimmune disease) were 0.47 (95% CI, 0.12–1.87) for breast cancer, 1.05 (95% CI, 0.15–7.49) for endometrial and 1.09 (95% CI, 0.62–1.93) for other female genital cancers. Adjustments for multiple confounders (e.g., sociodemographic factors, reproductive and gynecologic history, and proxy for life habits) were made. 

A cross-sectional study used agglomerated data from the John Hopkins hospital records to compare morphea patients with the general pool of patients between 2012 and 2018. Morphea patients were more likely to be diagnosed with melanoma with an OR of 6.6 (95% CI, 4.1–10.9), SCC (OR 12.8; 95% CI, 4.1–10.9) and BCC (OR 13.1; 95% CI, 9.7–17.5) compared to the control group [14]. The risk of developing skin cancer in morphea patients remained after adjusting for race. However, this study was cross-sectional and hence the temporal relationship was not established. Adjustment for other confounders such as age, sex and history of sun exposure was not performed. 

### 3.2. The Risk of Cancer in EF Patients

Seven articles reported on a new onset of cancer (3 or more years after diagnosis) in EF patients. All the articles were case reports (7/7). All EF cases were confirmed by histopathology. EF was limited to the extremities in 4/7 patients and generalized in 3/7 patients. Four patients were females (57.1%). The data on race were lacking. The mean age at cancer diagnosis was 69 years old (SD, 7.7, [*n* = 7]). The mean time to malignancy after EF onset was 3.9 years (SD, 15.7, [*n* = 7]). Hematological malignancies were seen in 5/7 patients (71.4%), 1 patient developed a solid organ malignancy, and another patient had a non-melanoma skin cancer. 

### 3.3. Paraneoplastic Morphea

Paraneoplastic morphea (defined as cancer onset concomitant to or within 3 years of diagnosis) was reported in 16 case reports, totaling 18 patients. Two-thirds of patients (12/18 or 66.7%) were females, and most were White (8/9 or 88.9%). The mean age at cancer diagnosis was 56.1 years old (SD, 18.1, [*n* = 10]). The mean time between morphea onset and cancer diagnosis was 1.0 year (SD, 1.0, [*n* = 14]). Generalized morphea was the most frequent morphea subtype (11/18 or 61.1%), followed by circumscribed (5/18 or 27.8%) and linear (2/18 or 11.1%). There was no predilection to a specific cancer. Breast cancer was seen in three patients, followed by rectal (2/18 or 11.1%) and single cases of odontogenic, lung, colon, prostate, bladder, cholangiocarcinoma, and endometrial carcinomas. There were six cases of hematological malignancies. 

### 3.4. Paraneoplastic EF

Twenty-nine articles including 48 EF patients were identified. Of these, 26/29 (89.7%) were case reports and 3/29 (10.3%) were observational studies. In most cases (37/48 or 77.1%), EF was confirmed by histopathology. EF was localized to the extremities in 70.0% of patients (21/30) and involved the entire body in 30.0% (9/30). Similar sex distribution was seen (16/31 or 51.6% females). Race was only reported in six cases (three were White and three were Asian). Cancer was diagnosed at a mean age of 63.0 years old (SD, 14.5, [*n* = 27]). Mean time between EF and cancer diagnoses was 10 months (SD, 12.8, [*n* = 38]). 

Hematological malignancies were seen in 62.5% of cases (30/48), followed by solid malignancies (16/48 or 33.3%), one patient developed both solid and hematological malignancies and one patient had non-melanoma skin cancer. 

Among the three observational studies, one was cross-sectional and two were retrospective single center. A cross-sectional study conducted in the Netherlands reported that 2/35 patients developed paraneoplastic EF. These malignancies were prostate carcinoma and intestinal carcinoid tumour. However, the timing between the two diagnoses was not mentioned [25].

Two retrospective chart reviews were conducted at the Mayo Clinic. The first study published in 1988 included 52 EF patients and reported that 7.7% of EF patients had an associated malignancy. Precisely, three were diagnosed with hematological malignancy within 1 year of EF and one with breast carcinoma (EF resolved spontaneously following mastectomy) [26]. The second study reported a 13.4% risk of associated malignancy among 89 EF patients diagnosed from 1997 to 2016. Of these, six were hematologic and six were solid tumors [27].

### 3.5. The Risk of Developing Morphea in Patients Following Cancer Therapy 

One hundred and five articles included 212 patients who developed morphea as a result of cancer treatment. Of these, 93 were case reports (93/105 or 88.6%), 9 case series (9/105 or 8.6%) and 3 observational studies (3/105 or 2.9%). The diagnosis of morphea was confirmed by histopathology in 89.6% of cases (129/144). When reported, most patients were females (193/208 or 92.8%) and White (48/54 or 88.9%). There were no reported juvenile cases. Mean age at cancer diagnosis was 57.8 (SD, 12.1, [*n* = 124]) years old. The most common cancers were breast (169/212 or 79.7%), followed by melanoma (12/212 or 5.7%) and lung cancer (12/212 or 5.7%).

Most morphea cases occurred after radiation therapy (138/212 or 65.0%) followed by chemotherapy (18/212 or 8.5%) and immunotherapy with immune checkpoint inhibitors (ICIs) (12/212 patients or 5.7%), whereas surgery induced morphea in one patient. The type of morphea is described in Table 1 according to cancer therapy. In 20.3% of patients (43/212), morphea occurred in the context of a multimodal cancer treatment where radiotherapy and/or chemotherapy/targeted agents and/or ICIs were combined. Table S2 presents multimodal cancer treatments. The mean time from treatment to morphea onset was 3.1 years for radiotherapy, 1.6 years for chemotherapy and 0.8 years for ICIs (Table 1). Pemetrexed (7/18 or 38.9%) and docetaxel (5/18 or 27.8%) were the most common chemotherapeutic agents associated with morphea whereas nivolumab (6/12 or 50.0%) and pembrolizumab (4/12 or 33.3%) were the most common ICIs. Of interest, no published cases were identified following CTLA-4 inhibitors. 

Among nine case series published, several articles attempted to estimate the incidence of radiation-included morphea among all patients undergoing radiotherapy by using as denominator the approximate number of patients they believed they had seen in their department with incidence rates varying from 0.025% to 0.26% [28,29,30]. While these incidence rates were frequently recited in the literature, they should be interpreted with caution as the real denominator was not known in either of the studies. We identified only one study (a retrospective cohort from Austria) that assessed the cumulative incidence of radiation-induced morphea among 2268 breast cancer patients between 2009 and 2018. Six patients developed morphea with a cumulative incidence of 0.26% (95% CI; 0.24–0.28), which equals one morphea case per 378 post-radiated patients [31]. In all cases, morphea was localized to the breast (either unilateral or bilateral) and appeared 3–10 months following radiation. No association with radiation parameters was seen. 

A multicenter retrospective chart review on radiation-induced morphea was conducted at Yale New Haven Hospital, Northwestern Memorial Hospital and Stanford University between 2000 and 2018. The medical record search yielded 25 cases. Most were females (23/25 or 92.0%) with invasive ductal carcinoma (19/25 or 76.0%). The mean latency period from radiation treatment was 35.1 months. Eleven patients (11/25 or 44.0%) had coexisting autoimmune diseases (six patients had rheumatoid arthritis). Over 56% (14/25) of patients developed lesions outside of the radiation field. Among these 14 patients, 64.3% (9/14) had pre-existing autoimmunity. Among 10 patients with severe radiation-induced morphea, risk factors that correlated with disease severity were autoimmunity, smoking history, and breast implantation [32]. 

### 3.6. The Risk of Developing EF in Patients Following Cancer Therapy

Twenty articles reported the development of EF in patients who had previously received cancer treatment. Of these, 19 were case reports (95.0%), and 1 (5.0%) was an observational study. In total, 24 EF patients were included. In 79.2% of cases (19/24), the diagnosis was confirmed by histopathology. Most cases were limited to the extremities (14/24 or 58.3%), followed by the entire body (6/24 or 25.0%) and the trunk (2/24 or 8.3%). Thirteen patients (54.2%) were female. Among five reported cases, all patients were White. Mean age at cancer diagnosis was 56.8 years old (SD, 14.8, [*n* = 14]). Mean time to EF onset after treatment initiation was 11.5 months (SD, 6.1, [*n* = 21]).

Reported cancers were predominantly solid malignancies (20/24 or 83.3%), followed by non-melanoma skin cancers (2/24, 8.3%) and only one hematological malignancy (1/24 or 4.2%). The most common drugs associated with EF development were ICIs (17/24 or 70.8%). Specifically, four cases occurred after nivolumab, four after pembrolizumab, three following a combination of ICIs, four after combination of ICIs and chemotherapy and two with ICIs and radiotherapy. As for morphea, no cases were identified due to CTLA-4 inhibitors as monotherapy. Only two patients developed EF after radiation therapy and two after receiving tamoxifen. 

An observational study from Israel published in 2018 reported rheumatic manifestations in 14/400 patients treated with ICIs. Of these, one patient developed EF after 8 months of pembrolizumab for melanoma [33].

## 4. Discussion

In this scoping review, we aimed to address the association between morphea/EF and malignancy. Specifically, we sought to answer the following questions: (1) Are patients with morphea/EF at an increased risk of malignancy? (2) Can morphea/EF present as a paraneoplastic phenomenon? (3) What are the prevalence and the culprits of morphea/EF arising due to cancer therapy? 

We identified 201 articles looking at the association between morphea/EF in cancer. Specifically, 32 papers (338/640 patients or 52.8%) focused on the risk of developing malignancies secondary to morphea/EF, 45 (66/640 or 10.3% patients) looked at morphea/EF as a paraneoplastic phenomenon and 125 (236/640 or 36.9% patients) described morphea/EF arising secondary to cancer therapy. 

The data regarding the risk of developing secondary malignancy in morphea/EF patients are limited. In morphea, only three observational studies were identified looking specifically for breast/gynecologic malignancies, pancreatic cancer, and skin cancer. Overall, there was no evidence to suggest an increased risk of breast/gynecologic malignancies. However, a ~30% increased risk of pancreatic cancer was suggested. Similarly, an increased risk of melanoma (OR ~6) and non-melanoma skin cancers (OR up to 13) was documented in one study. When looking at the patient characteristics of who developed cancer, generalized and pansclerotic morphea comprised over ⅔ of patients. Fortunately, among 331 morphea patients who developed cancer, only two patients developed cancer during adolescence and none during childhood. While the mean age at cancer diagnosis was 38.8 years, there are not enough data to suggest that morphea patients may be diagnosed with cancer at a younger age. In EF, only seven case reports of subsequent malignancy were identified. 

An increased risk of cancer has indeed been documented in many other systemic autoimmune rheumatic diseases such as systemic lupus erythematosus and SSc [34,35]. The risk is likely multifactorial, driven by chronic inflammation, oxidative stress, decreased immune surveillance, the use of systemic immunosuppressive therapies and external triggers associated with these conditions (e.g., silica or solvents in context of SSc) [18,36,37]. Chronic fibrotic diseases (e.g., morphea, SSc and scars) may carry an additional risk due to fibrogenesis and disruption of normal tissue architecture, creating favorable conditions for the growth and spread of cancer cells [38,39,40]. However, besides the development of SCC in chronic scars (i.e., Marjolin’s ulcer), whether skin cancer predominantly affects fibrotic skin in morphea patients’ needs further research. Hence, it is plausible that the most severe forms of morphea are particularly at risk of cancer [41]. While additional data are needed to address the risk of cancer in morphea/EF patients, we believe that a total body skin examination is warranted to monitor for skin cancer development during the long-term follow-up of morphea patients, particularly with more severe phenotypes. There are insufficient data to recommend additional screening, hence it should be guided by clinical history and individual risk factors. 

Contrary to the observation of more morphea patients being at an increased risk of developing cancer compared to EF patients, an inverse finding was observed regarding morphea/EF presenting as a paraneoplastic phenomenon. Paraneoplastic morphea was anecdotally reported in 18 patients, 11 of whom had a generalized subtype. On the other hand, there are more data regarding paraneoplastic EF, which occurs in ~10% of all EF patients, most often in association with hematologic malignancies [26,27,42,43]. The pathogenesis of paraneoplastic EF remains unknown. However, some authors hypothesized a shared immune dysregulation and neoantigen-induced T-cell response with type 2 skewing (e.g., IL-3/5 cytokines, eosinophilia) [44,45]. However, additional studies are required to confirm the incidence of paraneoplastic EF and elucidate the associated malignancies, clinical characteristics of patients at risk and its pathogenesis. For now, we believe that a narrow workup for hematologic malignancy would be prudent in all patients (i.e., complete blood count, smear, lactate dehydrogenase, protein electrophoresis), and further paraneoplastic workup may be guided by complete history and physical examination. 

While most of the studies identified in this scoping review focused on the development of morphea and EF following cancer therapy, only one study looked at the cumulative incidence focusing on radiation-induced morphea in breast cancer patients, with incidence being 0.26% [31]. Additional studies attempted to estimate the incidence of radiation-induced morphea. However, they used case series as a design without a precise denominator, but their incidence varied similarly between 0.025% to 0.26% [28,29,30]. While the cumulative incidence of post-radiation morphea is low, it is >100 times higher than the cumulative lifetime prevalence estimates in the general population (200 per 100,000 people) [1,2]. There was no incidence or prevalence data regarding the risk of morphea following other cancer therapies, such as chemotherapy, hormonal therapy or ICIs. The mean latency period for morphea varied according to the type of cancer treatment, with a trend for faster onset following ICIs.

Two-thirds of morphea cases reported were induced by radiotherapy, with a mean time to morphea onset of 3.2 years. Timing may help to distinguish morphea from radiation dermatitis, which appears within the first weeks of treatment and is considered an acute side effect [46]. Most radiation-induced cases were localized to the radiation field and were circumscribed morphea. However, in patients with a history of autoimmunity (e.g., rheumatoid arthritis or positive ANA antibodies), morphea beyond the radiation field was common. Chemotherapy-induced morphea was reported in 14 patients with a mean time to morphea onset of 1.7 years. In half of the cases, morphea was generalized, pansclerotic or mixed. A commonly described scenario was a patient with symmetric bilateral limb involvement (e.g., generalized symmetric morphea subtype). With immunotherapy-induced morphea, the mean time to morphea onset was 8 months, which indicates a rapid manifestation of morphea after ICIs initiation. All of the immunotherapy-induced cases were reported with programmed cell death protein-1 (PD-1) inhibitors and generally had similar clinical features (demographics, subtype) to idiopathic morphea. 

Most EF cases were triggered by ICIs (70.8% of patients) and similarly occurred following PD-1 inhibitors (e.g., nivolumab and pembrolizumab). It is unknown why all cases of morphea/EF were related to PD-1 blockers, and none occurred secondary to CTLA-4 inhibition. However, as CTLA-4 and PD-1 inhibitors target different checkpoints in the immune system, this difference could contribute to morphea/EF onset. CTLA-4 inhibitors block CTLA protein, thereby attenuating inhibition of early T cell activation [47]. PD-1 inhibitors, on the other hand, target the PD-1 protein expressed primarily on the surface of activated effector T-cells in peripheral tissues [47]. Therefore, the phase at which these ICIs mostly intervene in the immune response may explain the difference observed in the onset or absence of morphea/EF [47]. This should be further studied. 

Our study presents several limitations that are inherent to the nature of a scoping review. As scoping reviews prioritize mapping the literature rather than conducting detailed epidemiological analyses, this limits our ability to draw definitive epidemiological conclusions. Another inherent limitation is the lack of quality assessment for the included studies. This limitation restricts our ability to evaluate the methodological rigour or risk of bias in our included studies. Moreover, despite our efforts to contact authors twice when the articles were unavailable, a few articles could not be obtained, which introduces the possibility of missing eligible studies. Despite this, we believe we were able to capture most of the available literature to date through a comprehensive search across multiple search engines, using a rigorous and inclusive search strategy. Some articles had missing or incomplete information. While we reported the missing data to ensure transparency, missing data could influence the results presented. Finally, most of the included studies were case reports and case series with inherent publication bias. To mitigate the impact of this potential bias, we presented the characteristics of morphea/EF from all study types but focused more on data derived from observational studies.

## 5. Conclusions

In conclusion, we determined that to date, there is not enough evidence to suggest paraneoplastic morphea. However, morphea patients (particularly severe subtypes) may develop subsequent skin and possibly pancreatic cancer. Therefore, we recommend a total body skin examination to monitor skin cancer development during the long-term follow-up of morphea patients, particularly with more severe subtypes. However, additional research is needed to clarify the risk of pancreatic cancer prior to drawing conclusions. There are insufficient data to suggest a higher risk of future malignancies in EF, but paraneoplastic EF was seen in approximately 10% of cases, with hematologic cancers representing two-thirds of malignancies. A narrow workup for hematologic malignancies may be prudent. While further data are needed to confirm a higher risk of cancer in EF, additional workup may be guided by clinical history and individual risk factors. Both morphea and EF were reported to occur following cancer therapy. Most data in morphea occurred in the context of radiation therapy followed by chemotherapy and ICIs, whereas most EF cases were induced by ICIs. Of interest, no cases were reported due to CTLA-4 inhibition, suggesting a differential risk of developing morphea/EF with PD-1 inhibitors. Additional research (e.g., population-based and/or longitudinal observational studies) studying the association between malignancy and morphea/EF is needed.

## Figures and Tables

**Table 1:** Type of Morphea According to Cancer Treatment.

	Radiotherapy (%)	Chemotherapy (%)	Surgery (%)	Immunotherapy (%)	Multimodal Treatment (%)
Generalized	15 (11)	12 (67)	1 (100)	4 (22)	21 (49)
Circumscribed	120 (87)	6 (33)	0	6 (50.0)	21 (49)
Pansclerotic	0	0	0	0	0
Linear	2 (1)	0	0	0	1 (2)
Mixed	1 (<1)	0	0	0	0
Unknown	0	0	0	2 (17)	0
Total Number of Patients (%)	138	18	1 (<1)	12	43
Mean time to morphea onset from cancer treatment (years)	3.1 (SD, 4.6, [*n* = 99])	1.6 (SD, 3.0, [*n* = 10])	-	0.8 (SD, 0.4, [*n* = 10])	2.4 (SD, 3.1, [*n* = 35])

## Data Availability

The data can be shared up on request.

## Supplementary Materials

The following supporting information can be downloaded: https://www.mdpi.com/article/10.3390/cancers15184450/s1, Supplementary Materials: Search strategy; Table S1: Articles reporting localized scleroderma or eosinophilic fasciitis with malignancy; Table S2: Multimodal cancer treatment.

## References

[1] Mahmood F., Nguyen A., Muntyanu A., Jfri A., McCuaig C., Chédeville G., Piram M., Netchiporouk E. (2022). Prevalence and Incidence of Localized Scleroderma: A Qualitative Systematic Review. J. Cutan. Med. Surg..

[2] Lagacé F., D’Aguanno K., Prosty C., Laverde-Saad A., Cattelan L., Ouchene L., Oliel S., Genest G., Doiron P., Richer V. (2023). The Role of Sex and Gender in Dermatology—From Pathogenesis to Clinical Implications. J. Cutan. Med. Surg..

[3] Grabell D., Hsieh C., Andrew R., Martires K., Kim A., Vasquez R., Jacobe H. (2014). The Role of Skin Trauma in the Distribution of Morphea Lesions: A Cross-Sectional Survey of the Morphea in Adults and Children Cohort IV. J. Am. Acad. Dermatol..

[4] Jacobe H., Ahn C., Arnett F.C., Reveille J.D. (2014). Major Histocompatibility Complex Class I and Class II Alleles May Confer Susceptibility to or Protection against Morphea: Findings from the Morphea in Adults and Children Cohort. Arthritis Rheumatol..

[5] Tomimura S., Ogawa F., Iwata Y., Komura K., Hara T., Muroi E., Takenaka M., Shimizu K., Hasegawa M., Fujimoto M. (2008). Autoantibodies against Matrix Metalloproteinase-1 in Patients with Localized Scleroderma. J. Dermatol. Sci..

[6] Higley H., Persichitte K., Chu S., Waegell W., Vancheeswaran R., Black C. (1994). Immunocytochemical Localization and Serologic Detection of Transforming Growth Factor Beta 1. Association with Type I Procollagen and Inflammatory Cell Markers in Diffuse and Limited Systemic Sclerosis, Morphea, and Raynaud’s Phenomenon. Arthritis Rheum..

[7] Leitenberger J.J., Cayce R.L., Haley R.W., Adams-Huet B., Bergstresser P.R., Jacobe H.T. (2009). Distinct Autoimmune Syndromes in Morphea: A Review of 245 Adult and Pediatric Cases. Arch. Dermatol..

[8] Mertens J.S., Seyger M.M.B., Thurlings R.M., Radstake T.R.D.J., de Jong E.M.G.J. (2017). Morphea and Eosinophilic Fasciitis: An Update. Am. J. Clin. Dermatol..

[9] Giat E., Ehrenfeld M., Shoenfeld Y. (2017). Cancer and Autoimmune Diseases. Autoimmun. Rev..

[10] Igusa T., Hummers L.K., Visvanathan K., Richardson C., Wigley F.M., Casciola-Rosen L., Rosen A., Shah A.A. (2018). Autoantibodies and Scleroderma Phenotype Define Subgroups at High-Risk and Low-Risk for Cancer. Ann. Rheum. Dis..

[11] Landgren A.M., Landgren O., Gridley G., Dores G.M., Linet M.S., Morton L.M. (2011). Autoimmune Disease and Subsequent Risk of Developing Alimentary Tract Cancers among 4.5 Million US Male Veterans. Cancer.

[12] Makino K., Jinnin M., Hirano A., Yamane K., Eto M., Kusano T., Honda N., Kajihara I., Makino T., Sakai K. (2013). The Downregulation of microRNA Let-7a Contributes to the Excessive Expression of Type I Collagen in Systemic and Localized Scleroderma. J. Immunol..

[13] Kim S.W., Rice L., Champlin R., Udden M.M. (1997). Aplastic Anemia in Eosinophilic Fasciitis: Responses to Immunosuppression and Marrow Transplantation. Haematologia.

[14] Boozalis E., Shah A.A., Wigley F., Kang S., Kwatra S.G. (2019). Morphea and Systemic Sclerosis Are Associated with an Increased Risk for Melanoma and Nonmelanoma Skin Cancer. J. Am. Acad. Dermatol..

[15] Fiorentino D.F., Casciola-Rosen L. (2022). Autoantibodies and Cancer Association: The Case of Systemic Sclerosis and Dermatomyositis. Clin. Rev. Allergy Immunol..

[16] Fragoulis G.E., Daoussis D., Pagkopoulou E., Garyfallos A., Kitas G.D., Dimitroulas T. (2020). Cancer Risk in Systemic Sclerosis: Identifying Risk and Managing High-Risk Patients. Expert Rev. Clin. Immunol..

[17] Yasunaga M. (2020). Antibody Therapeutics and Immunoregulation in Cancer and Autoimmune Disease. Semin. Cancer Biol..

[18] Joseph C.G., Darrah E., Shah A.A., Skora A.D., Casciola-Rosen L.A., Wigley F.M., Boin F., Fava A., Thoburn C., Kinde I. (2014). Association of the Autoimmune Disease Scleroderma with an Immunologic Response to Cancer. Science.

[19] Ansari S., Iftikhar U., Jamil A., Ansari A., Iftikhar S. (2021). Eosinophilic Fasciitis With a Malignant Outcome. J. Med. Cases.

[20] Prasad S., Zhu J.L., Schollaert-Fitch K., Torok K.S., Jacobe H.T. (2021). An Evaluation of the Performance of Current Morphea Subtype Classifications. JAMA Dermatol..

[21] Abbas L., Joseph A., Kunzler E., Jacobe H.T. (2021). Morphea: Progress to Date and the Road Ahead. Ann. Transl. Med..

[22] Heck J., Olk J., Kneitz H., Hamm H., Goebeler M. (2020). Long-Standing Morphea and the Risk of Squamous Cell Carcinoma of the Skin. J. Dtsch. Dermatol. Ges..

[23] Yuan F., Pfeiffer R.M., Julián-Serrano S., Arjani S., Barrett M.J., Koshiol J., Stolzenberg-Solomon R.Z. (2023). Autoimmune Conditions and Pancreatic Cancer Risk in Older American Adults. Int. J. Cancer.

[24] Hemminki K., Liu X., Ji J., Försti A., Sundquist J., Sundquist K. (2012). Effect of Autoimmune Diseases on Risk and Survival in Female Cancers. Gynecol. Oncol..

[25] Mertens J.S., Thurlings R.M., Kievit W., Seyger M.M.B., Radstake T.R.D., de Jong E.M.G.J. (2017). Long-Term Outcome of Eosinophilic Fasciitis: A Cross-Sectional Evaluation of 35 Patients. J. Am. Acad. Dermatol..

[26] Lakhanpal S., Ginsburg W.W., Michet C.J., Doyle J.A., Moore S.B. (1988). Eosinophilic Fasciitis: Clinical Spectrum and Therapeutic Response in 52 Cases. Semin. Arthritis Rheum..

[27] Mango R.L., Bugdayli K., Crowson C.S., Drage L.A., Wetter D.A., Lehman J.S., Peters M.S., Davis M.D., Chowdhary V.R. (2020). Baseline Characteristics and Long-Term Outcomes of Eosinophilic Fasciitis in 89 Patients Seen at a Single Center over 20 Years. Int. J. Rheum. Dis..

[28] Ardern-Jones M.R., Black M.M. (2003). Widespread Morphoea Following Radiotherapy for Carcinoma of the Breast. Clin. Exp. Dermatol..

[29] Davis D.A., Cohen P.R., McNeese M.D., Duvic M. (1996). Localized Scleroderma in Breast Cancer Patients Treated with Supervoltage External Beam Radiation: Radiation Port Scleroderma. J. Am. Acad. Dermatol..

[30] Bleasel N.R., Stapleton K.M., Commens C., Ahern V.A. (1999). Radiation-Induced Localized Scleroderma in Breast Cancer Patients. Australas. J. Dermatol..

[31] Partl R., Regitnig P., Lukasiak K., Winkler P., Kapp K.S. (2020). Incidence of Morphea Following Adjuvant Irradiation of the Breast in 2,268 Patients. Breast Care.

[32] Mittal A., Mittal V., Panse G., Choi J.N., Kwong B.Y., Leventhal J.S. (2019). Radiation-Induced Morphea: Association with Autoimmune Comorbidities, Severity, and Response to Therapy. J. Am. Acad. Dermatol..

[33] Lidar M., Giat E., Garelick D., Horowitz Y., Amital H., Steinberg-Silman Y., Schachter J., Shapira-Frommer R., Markel G. (2018). Rheumatic Manifestations among Cancer Patients Treated with Immune Checkpoint Inhibitors. Autoimmun. Rev..

[34] Morrisroe K., Nikpour M. (2020). Cancer and Scleroderma: Recent Insights. Curr. Opin. Rheumatol..

[35] Maria A.T.J., Partouche L., Goulabchand R., Rivière S., Rozier P., Bourgier C., Le Quellec A., Morel J., Noël D., Guilpain P. (2018). Intriguing Relationships Between Cancer and Systemic Sclerosis: Role of the Immune System and Other Contributors. Front. Immunol..

[36] Muntyanu A., Milan R., Rahme E., LaChance A., Ouchene L., Cormier M., Litvinov I.V., Hudson M., Baron M., Netchiporouk E. (2022). Exposure to Silica and Systemic Sclerosis: A Retrospective Cohort Study Based on the Canadian Scleroderma Research Group. Front. Med..

[37] Hill C.L., Nguyen A.-M., Roder D., Roberts-Thomson P. (2003). Risk of Cancer in Patients with Scleroderma: A Population Based Cohort Study. Ann. Rheum. Dis..

[38] Aggarwal B.B., Sung B., Gupta S.C. (2014). Inflammation and Cancer.

[39] Piersma B., Hayward M.-K., Weaver V.M. (2020). Fibrosis and Cancer: A Strained Relationship. Biochim. Biophys. Acta Rev. Cancer.

[40] Belmesk L., Muntyanu A., Cantin E., AlHalees Z., Jack C.S., Le M., Sasseville D., Iannattone L., Ben-Shoshan M., Litvinov I.V. (2022). Prominent Role of Type 2 Immunity in Skin Diseases: Beyond Atopic Dermatitis. J. Cutan. Med. Surg..

[41] Moysidou E., Lioulios G., Xochelli A., Nikolaidou V., Christodoulou M., Mitsoglou Z., Stai S., Fylaktou A., Papagianni A., Stangou M. (2022). Different Types of Chronic Inflammation Engender Distinctive Immunosenescent Profiles in Affected Patients. Int. J. Mol. Sci..

[42] Chohan S., Wong N., Hanson J., Osto M., Daveluy S. (2023). Eosinophilic Fasciitis May Present as a Paraneoplastic Syndrome of Hematological Malignancies-A Systematic Review. JAAD Int..

[43] Haddad H., Sundaram S., Magro C., Gergis U. (2014). Eosinophilic Fasciitis as a Paraneoplastic Syndrome, a Case Report and Review of the Literature. Hematol. Oncol. Stem Cell Ther..

[44] French L.E., Shapiro M., Junkins-Hopkins J.M., Wolfe J.T., Rook A.H. (2003). Eosinophilic Fasciitis and Eosinophilic Cellulitis in a Patient with Abnormal Circulating Clonal T Cells: Increased Production of Interleukin 5 and Inhibition by Interferon Alfa. J. Am. Acad. Dermatol..

[45] Chan L.S., Hanson C.A., Cooper K.D. (1991). Concurrent Eosinophilic Fasciitis and Cutaneous T-Cell Lymphoma. Eosinophilic Fasciitis as a Paraneoplastic Syndrome of T-Cell Malignant Neoplasms?. Arch. Dermatol..

[46] Rosenthal A., Israilevich R., Moy R. (2019). Management of Acute Radiation Dermatitis: A Review of the Literature and Proposal for Treatment Algorithm. J. Am. Acad. Dermatol..

[47] Terrier B., Humbert S., Preta L.-H., Delage L., Razanamahery J., Laurent-Roussel S., Mestiri R., Beaudeau L., Legendre P., Goupil F. (2020). Risk of Scleroderma according to the Type of Immune Checkpoint Inhibitors. Autoimmun. Rev..

