# Supplementary material for: Morphea, Eosinophilic Fasciitis and Cancer: A Scoping Review
Source: Cancers (Basel). 2023 Sep 7;15(18):4450. doi: 10.3390/cancers15184450 (PMC10526289; doi:10.3390/cancers15184450)
Supplement: Supplementary file 1 [file cancers-15-04450-s001.zip › Supplementary_Material_Table_S1_morphea cancers_Revised_MJC.pdf]

Supplementary Material Table S1. Articles reporting morphea or eosinophilic fasciitis with malignancy.

Title	Medical Condition	Aim (1, 2, 3)	Authors	Published Year	Age at cancer diagnosis	Adress Reprint Country	Sex	Cancer type	Number of patients
Scleroderma and carcinoma	Morphea	1	FORMAN, L; ATKINS, H	1958	n/a	N/A	F	Rectum	1
Sclerodermiform aspect of arm lymphoedema after treatment with docetaxel for breast cancer.	Morphea	3	Vignes, S; Lebrun-Vignes, B	2007	44-65	N/A	11F	Breast	11
Scleroderma-like syndrome due to hydroxyurea.	Morphea	3	Garcia-Martinez, F J; Garcia-Gavin, J; Alvarez-Perez, A; Alonso-Gonzalez, J; Ginarte, M; Toribio, J	2012	45	Spain	M	Hematological	1
Glycosaminoglycan and versican deposits in taxane-induced sclerosis.	Morphea	3	Okada, K; Endo, Y; Miyachi, Y; Koike, Y; Kuwatsuka, Y; Utani, A	2015	56, 59	Japan	2M	Hematological	2
Pemetrexed-induced scleroderma-like conditions in the lower legs of a patient with non-small cell lung carcinoma.	Morphea	3	Ishikawa, Kazushi; Sakai, Takashi; Saito-Shono, Tomoko; Miyawaki, Michiyo; Osoegawa, Atsushi; Sugio, Kenji; Ono, Asami; Mori, Hiromu; Nishida, Haruto; Yokoyama, Shigeo; Okamoto, Osamu; Fujiwara, Sakuhei; Hatano, Yutaka	2016	n/a	Japan	M	Lung	1
Scleroderma-like syndrome associated with nivolumab treatment in malignant melanoma.	Morphea	3	Cho, Maria; Nonomura, Yumi; Kaku, Yo; Nakabo, Shuichiro; Endo, Yuichiro; Otsuka,	2019	n/a	Japan	M	Melanoma	1

			Atsushi; Kabashima, Kenji						
Squamous cell carcinoma and eosinophilia in a long-term course of pansclerotic morphea.	Morphea	1	Grewal, Inderjote; Khan, Omar; Davis, William	2014	25	USA	M	SCC	1
Squamous cell carcinoma arising from morphoea	Morphea	1	Anandan G.; Jayakumar J.; Aschhoff M.; Kurian S.; Job C.K.	1994	n/a	N/A	F	SCC	1
Morphoea associated with B-cell lymphoma	Morphea	2	Ishikawa O.; Akimoto S.; Iijima C.; Takeuchi Y.; Miyachi Y.; Nagai Y.; Kojima M.	1997	n/a	Japan	F	Hematological	1
Disseminated morphea in small cell lung cancer	Morphea	3	Benekli M.; Yalcin B.; Guler N.; Tekuzman G.	1998	34	N/A	F	Lung	1
Localized scleroderma in a woman irradiated at two sites for endometrial and breast carcinoma: a case history and a review of the literature	Morphea	3	Ulléen, Bjorkholm	2003	67	Sweden	F	Endometrial	1
Postirradiation morphea of the breast. Presentation of two cases and review of the literature	Morphea	3	Schaffer J.V.; Carroll C.; Dvoretsky I.; Huether M.J.; Girardi M.	2000	n/a	USA	2F	Breast	2
[Localized scleroderma as a manifestation of a cancer of the rectum]	Morphea	2	A Y Joliot, F Vittori, C Allard, R Levrat	1979	n/a	France	M	Rectum	1
Localized morphea after silicone-gel-filled breast implant	Morphea	3	Granel B.; Serratrice J.; Gaudy C.; Weiller-Merli C.; Bonerandi J.J.; Lepidi H.; Coulomb-Marchetti B.; Disdier P.; Weiller P.J.	2001	53	France	F	Breast	1
Postirradiation morphea in breast cancer	Morphea	3	Martin J.M.; Pinazo M.I.; Monteagudo C.;	2003	56	N/A	F	Breast	1

			Calduch L.; Ramon D.; Jorda E.						
Regression of morphoea plaques after tamoxifen therapy	Morphea	1	Ayoub N.; Bouaziz J.-D.; Barete S.; Frances C.	2004	59	N/A	F	Breast	1
Postirradiation morphea and subcutaneous polyarteritis nodosa: Case report and literature review	Morphea	3	Reddy S.M.; Pui J.C.; Gold L.I.; Mitnick H.J.	2005	75	USA	F	Breast	1
Systemic sclerosis, morphoea and breast cancer	Morphea	2	Mittal G.; Maddison P.; Williams W.	2006	n/a	North Wales	F	Breast	1
Morphea of the breast. Two case reports and discussion of the literature	Morphea	3	Dancey A.L.; Waters R.A.	2006	61	UK	F	Breast	1
Disabling pansclerotic morphea of childhood poses a high risk of chronic ulceration of the skin and squamous cell carcinoma	Morphea	1	Wollina U.; Buslau M.; Heinig B.; Petrov I.; Unger E.; Kyriopoulou E.; Koch A.; Kostler E.; Schonlebe J.; Haroske G.; Doede T.; Pramatarov K.	2007	16, 19	Germany	F, M	SCC	2
Generalized Morphea revealing rectal adenocarcinoma	Morphea	2	Masmoudi A.; Amouri M.; Khabir A.; Charfeddine A.; Salah H.B.; Sallemi M.; Amouri A.; Boudaya S.; Bouassida S.; Boudawara T.; Krichen M.; Daoud J.; Baklouti S.; Zahaf A.; Turki H.	2008	62	Turkey	F	Rectum	1
Localized Morphea - A Rare but Significant Secondary Complication Following Breast Cancer Radiotherapy : Case Report and Review of the Literature on Radiation	Morphea	3	Herrmann T.; Gunther C.; Csere P.	2009	n/a	Germany	F	Breast	1

Reaction among Patients with Scleroderma/Morphea									
Disabling pansclerotic morphoea: A case report	Morphea	1	Tekin N.S.; Altinyazar H.C.; Tekin I.O.; Keskin S.I.; Kucukoglu R.; Onsun N.	2010	27	Turkey	F	SCC	1
Postirradiation linear morphoea: Clinical dermatology * Concise report	Morphea	3	Akay B.N.; Sanli H.; Heper A.O.	2010	70	Turkey	F	Endometrial	1
Radiation-induced morphea (RIM)	Morphea	3	De Jager M.E.A.; De Jong E.M.G.J.; Kamsteeg M.	2014	n/a	N/A	N/A	Breast	1
Generalized morphea with development of bullous lesions at the site of surgical scars	Morphea	3	Martinez S.B.; Gargallo V.; Romero F.T.; Martinez L.A.; Peralto J.L.R.; Martin R.L.	2016	n/a	Spain	F	Breast	1
Post-irradiation morphea of the breast: does this pose an issue for reconstruction?	Morphea	3	Rafique B.; McInerney N.; Fitzgerald G.; O'Hanlon D.; Gilmore J.; Kelly E.J.	2017	48-57	Ireland	3F	Breast	3
Cutaneous paraneoplastic manifestation (Morphea, Lichen Sclerosus) - Two case reports	Morphea	2	Pappova T.; Pec J.; Kozarova A.; Adamicova K.	2017	54	Poland	F	Breast	1
Drug-induced localised scleroderma	Morphea	3	Maya Y.; Ota M.	2018	n/a	Japan	F	Breast	1
Morphea associated with primary biliary cirrhosis and Waldenstrom macroglobulinemia: Response to rituximab	Morphea	2	Traboulsi D.; Kaminska E.A.; Barr S.G.; Hunter C.; Mydlarski P.R.	2018	54	Canada	F	Hematological	1
Treatment of generalized deep morphea and eosinophilic fasciitis with the Janus kinase inhibitor tofacitinib	Morphea	3	Kim S.R.; Charos A.; Damsky W.; Heald P.; Girardi M.; King B.A.	2018	n/a	USA	F	Breast	1

Post-irradiation morphoea of the breast: a case report and review of the literature	Morphea	3	Gonzalez-Ericsson P.I.; Estrada M.V.; Al-Rohil R.; Sanders M.E.	2018	42	USA	F	Breast	1
Post radiotherapy deep morphea. A case report	Morphea	3	Corball M.V.; Virginia Garcia M.; Garay I.; Kurpis M.; Ruiz-Lascano A.	2020	n/a	Argentina	F	Breast	1
Postirradiation morphea: A case report with a review of the literature and summary of the clinicopathologic differential diagnosis	Morphea	3	Morganroth P.A.; DeHoratius D.; Curry H.; Elenitsas R.	2010	45	USA	F	Breast	1
Diffuse morphoea with calcinosis cutis and squamous-cell carcinoma.	Morphea	1	Michalowski, R	1967	31	Poland	M	SCC	1
B-cell markers in malignant B-cell lymphoma with scleroderma-like manifestation.	Morphea	2	Van Joost, T; Stolz, E; Blog, F B; Van der Kwast, T H; Vuzevski, V D; Van Dongen, J M	1984	n/a	N/A	F	Hematological	1
Post-irradiation morphoea.	Morphea	3	Colver, G B; Rodger, A; Mortimer, P S; Savin, J A; Neill, S M; Hunter, J A	1989	85	UK	9F	Hematological, breast, endocervix	9
Circumscribed scleroderma induced by postlumpectomy radiation therapy.	Morphea	3	Trattner, A; Figer, A; David, M; Lurie, H; Sandbank, M	1991	n/a	Israel	F	Breast	1
Vitiligo-like depigmentation and morpheas after specific intralymphatic immunotherapy for malignant melanoma.	Morphea	3	Lacour, J P; Caldani, C; Thyss, A; Schneider, M; Ortonne, J P	1992	66	France	F	Melanoma	1
Squamous cell carcinoma in localized scleroderma following immunosuppressive therapy with azathioprine.	Morphea	1	Nachbar, F; Stolz, W; Volkenandt, M; Meurer, M	1993	40	Germany	F	SCC	1

Bullous morphea: a distinct entity?.	Morphea	3	Trattner, A; David, M; Sandbank, M	1994	57	Israel	F	Breast	1
Docetaxel (Taxotere) associated scleroderma-like changes of the lower extremities. A report of three cases.	Morphea	3	Battafarano, D F; Zimmerman, G C; Older, S A; Keeling, J H; Burris, H A	1995	46-67	USA	2M, 1F	Leiomyosarcoma, melanoma, lung	3
Localized scleroderma in breast cancer patients treated with supervoltage external beam radiation: radiation port scleroderma.	Morphea	3	Davis, D A; Cohen, P R; McNeese, M D; Duvic, M	1996	n/a	USA	6F	Breast	6
[Deep morphea-type lesions, first manifestations of lymphocytic lymphoma].	Morphea	2	Plantin, P; Le Leannec, N; Delmas, A; Le Berre, A; Leroy, J P	1996	n/a	France	M	Hematological	1
Disseminated morphea in small cell lung cancer.	Morphea	2	Benekli, M; Yalcin, B; Guler, N; Tekuzman, G	1998	34	Norway	F	Lung	1
Postradiation morphea.	Morphea	3	Gollob, M H; Dekoven, J G; Bell, M J; Assaad, D; Rao, J	1998	n/a	Canada	F	Breast	1
Radiation-induced localized scleroderma in breast cancer patients.	Morphea	3	Bleasel, N R; Stapleton, K M; Commens, C; Ahern, V A	1999	38-60	Australia	4F	Breast	4
Squamous cell carcinoma arising in a patient with long-standing pansclerotic morphea.	Morphea	1	Parodi, P C; Riberti, C; Draganic Stinco, D; Patrone, P; Stinco, G	2001	20	Italy	F	SCC	1
Squamous cell carcinoma in pansclerotic morphea of childhood.	Morphea	1	Wollina, Uwe; Buslau, Michael; Weyers, Wolfgang	2002	16	Germany	M	SCC	1
Scleroderma-like cutaneous lesions induced by paclitaxel: a case study.	Morphea	3	Kupfer, I; Balguerie, X; Courville, P; Chinet, P; Joly, P	2003	63	France	F	Peritoneal cancer	1
Widespread morphoea following radiotherapy for carcinoma of the breast.	Morphea	3	Ardern-Jones, M R; Black, M M	2003	60	UK	F	Breast	1

Guttate morphoea in human T-cell lymphoma/lymphotrophic virus type-1 (HTLV-1) infection.	Morphea	2	Oiso, N; Fukai, K; Hosomi, N; Ishii, M	2003	n/a	Japanese	M	Hematological	1
Regression of morphoea plaques after tamoxifen therapy.	Morphea	3	Ayoub, N; Bouaziz, J-D; Barete, S; Frances, C	2004	59	Germany	F	Breast	1
From circumscribed scleroderma (morphea) to subcutaneous panniculitis-like T-cell lymphoma: case report.	Morphea	2	Troskot, Nina; Lugovic, Liborija; Situm, Mirna; Vucic, Majda	2004	n/a	Croatia	M	Hematological	1
Postirradiation morphea in a breast cancer patient.	Morphea	3	Dubner, Steve; Bovi, Joseph; White, Julia; Susnik, Barbara	2006	52	USA	F	Breast	1
Breast cancer and scleroderma.	Morphea	3	Scope, Alon; Sadetzki, Siegal; Sidi, Yechezkel; Barzilai, Aviv; Trau, Henry; Kaufman, Bela; Catane, Rafael; Ehrenfeld, Micahel	2006	43, 51	Israel	2F	Breast	2
Postirradiation morphea: an underrecognized complication of treatment for breast cancer.	Morphea	3	Walsh, Noreen; Rheaume, Dorianne; Barnes, Penelope; Tremaine, Robert; Reardon, Michael	2008	51-69	Canada	5F	Breast	5
Lower lip squamous cell carcinoma in disabling pansclerotic morphea of childhood.	Morphea	1	Petrov, Ivaylo; Gantcheva, Mary; Miteva, Ljubka; Vassileva, Snejina; Pramatarov, Kyrill	2009	19	Bulgaria	F	SCC	1
Morphea following surgery and radiotherapy: an evolving problem.	Morphea	3	Mosterd, K; Winnepenninckx, V; Vermeulen, A; van Neer, P A F A; van Neer, F J M A; Frank, J	2009	52	Neatherlands	F	Breast	1

Unrecognized radiation-induced localized scleroderma: a cause of postoperative wound-healing disorder.	Morphea	3	Kreft, B; Wohlrab, J; Radant, K; Danz, B; Marsch, W C; Fiedler, E	2009	n/a	UK	M	Gastric stump carcinoma	1
Postirradiation linear morphoea.	Morphea	3	Akay, B N; Sanli, H; Heper, A O	2010	74	Turkey	F	Endometrial	1
Synchronous angiosarcoma, melanoma and morphea of the breast skin 14 years after radiotherapy for mammary carcinoma.	Morphea	3	de Giorgi, Vincenzo; Santi, Raffaella; Grazzini, Marta; Papi, Federica; Gori, Alessia; Rossari, Susanna; Massi, Daniela; Lotti, Torello	2010	54	Italy	F	Breast	1
Scleroderma-like cutaneous lesions induced by paclitaxel and carboplatin for ovarian carcinoma, not a single course of carboplatin, but re-induced and worsened by previously administrated paclitaxel.	Morphea	3	Konishi, Yoshitomo; Sato, Hirokazu; Sato, Naoki; Fujimoto, Toshio; Fukuda, Jun; Tanaka, Toshinobu	2010	67	Japan	F	Ovarian	1
Cutaneous squamous cell carcinoma arising within generalised morphea.	Morphea	1	Saleh, Daniel B; Williams, Andrew M; Smith, Ian M	2011	59	UK	F	SCC	1
Morphea as a consequence of accelerated partial breast irradiation.	Morphea	3	Wernicke, A Gabriella; Goltser, Yevgeniya; Trichter, Samuel; Sabbas, Albert; Gaan, Jalong; Swistel, Alexander J; Magro, Cynthia M	2011	65	USA	F	Breast	1
Taxane-induced morphea in a patient with CREST syndrome.	Morphea	3	Bouchard, Susan M; Mohr, Melinda R; Pariser, Robert J	2010	73	USA	F	Breast	1
Radiation-induced morphea of the breast: a case report.	Morphea	3	Cheah, Nellie L C; Wong, Daniel W Y;	2008	57	UK	F	Breast	1

			Chetiyawardana, Anula D						
Post-Irradiation Morphea in Breast Cancer: An Uncommon Differential Diagnosis to Keep in Mind.	Morphea	3	Afonso-Afonso, Francisco Javier; Arevalo, Maria Pilar; Cerecedo, Fernando Campo; De Paz Arias, Laura; Tonder, Cristina Durana	2010	51	Spain	F	Breast	1
Generalized morphea after breast cancer radiation therapy.	Morphea	3	Kushi, Jonathan; Csuka, M E	2011	n/a	USA	F	Breast	1
Post-Irradiation Morphea: Case report and review of the literature.	Morphea	3	Alhathlool, Ammar; Hein, Rudiger; Andres, Christian; Ring, Johannes; Eberlein, Bernadette	2012	n/a	Germany	F	Breast	1
Success treatment of post-irradiation morphoea with acitretin and narrowband UVB.	Morphea	3	Newland, Kate; Marshman, Gillian	2012	43	Australia	F	Breast	1
Effect of autoimmune diseases on risk and survival in female cancers.	Morphea	1	Hemminki, Kari; Liu, Xiangdong; Ji, Jianguang; Forsti, Asta; Sundquist, Jan; Sundquist, Kristina	2012	N/A	Sweden	53F	Breast, endometrial, vagina	53
[Post-irradiation morphea in brest cancer: a case report].	Morphea	3	Llenas, Jorge; Bringas, Anahi; Nocito, Jimena; Gomez Zanni, Susana; Campana, Ricardo; Papa, Mariana	2012	n/a	Argentina	F	Breast	1
Generalized Morphea following Radiotherapy for an Intracranial Tumor.	Morphea	3	Balegar, Shrenik; Mishra, Dharmendra Kumar; Chatterjee, Sagarika; Kumari,	2016	27	India	M	Brain	1

			Shweta; Tiwary, Anup Kumar						
Focal seizures secondary to cortical dysplasia associated with isolated oral morphea and odontogenic carcinoma.	Morphea	2	McNamara, Patricia H; Toner, Mary; Kearns, Gerard; Keohane, Catherine; Daly, Peter; Doherty, Colin P	2013	16	Ireland	M	odontogenic carcinoma.	1
Late-onset en coup de sabre of the skull.	Morphea	2	Mohan, Shaun V; Nittur, Vinay; Stevens, Kathryn J	2013	62	USA	F	Breast	1
Radiation-induced morphoea treated with UVA-1 phototherapy.	Morphea	3	Lim, D; Johnston, S; Novakovic, L; Fearfield, L	2014	40	UK	F	Breast	1
Two cases of dermatoses koebnerizing within fields of previous radiotherapy.	Morphea	3	Hellen, R; Kiely, C; Murad, A; Mulligan, N; Coffey, J; Lenane, P; Moloney, F J	2014	53	Ireland	F	Breast	1
[Squamous cell carcinoma in localized scleroderma].	Morphea	1	Durcanska, V; Jedlickova, H; Slama, O; Velecky, L; Brezinova, E; Vasku, V	2014	26	Czech Republic	F	SCC	1
Morphea following radiation therapy in a patient with breast cancer.	Morphea	3	Garcia-Arpa, M; Lozano-Martin, E; Rodriguez, C Ramos; Rodriguez-Vazquez, M	2015	n/a	Spain	F	Breast	1
A case of radiation-induced generalized morphea with prominent mucin deposition and tenderness.	Morphea	3	Yanaba, Koichi; Umezawa, Yoshinori; Nakagawa, Hidemi	2015	n/a	Japan	F	Breast	1
Abdominopelvic post-irradiation morphea in a prostate cancer patient: the first case of an under-recognized condition.	Morphea	3	di Meo, Nicola; Noal, Cecilia; Trevisini, Sara; Ulessi, Bruno; Trevisan, Giusto	2015	n/a	Italy	M	Prostate	1

Paraneoplastic Scleroderma: Are There Any Clues?.	Morphea	2	Jedlickova, Hana; Durcanska, Veronika; Vasku, Vladimir	2016	66, 74	Czech Republic	2F, 1M	Cholangiogenic carcinoma, endometrial, prostatic adenocarcinoma, and adenoma of the suprarenal gland	3
Localized Morphea Developing in a Scar After Breast Carcinoma Surgery in the Absence of Radiotherapy.	Morphea	3	Chiriac, Anca; Podoleanu, Cristian; Coros, Marius F; Moldovan, Cosmin; Stolnicu, Simona	2016	n/a	Canada	F	Breast	1
Generalized morphea as the first sign of breast carcinoma: a case report.	Morphea	2	Desmond, Bryce L; Blattner, Collin M; Young Iii, John	2016	56	USA	F	Colon	1
Radiation-Induced Morphea: An Under-Recognized Complication of Breast Irradiation.	Morphea	3	Dyer, Brandon A; Hodges, Megan G; Mayadev, Jyoti S	2016	68, 70	USA	2F	Breast	2
Radiation-induced circumscribed superficial morphea after brachytherapy for endometrial adenocarcinoma.	Morphea	3	Trivedi, Apoorva; DeWitt, Corey M; McGevna, Laura	2017	n/a	USA	F	Endometrial	1
Hemo-Lymphopoietic Malignancies Surround the Women of the Family: A Case Report and Literature Review.	Morphea	1	Kalantari-Khandani, Behjat; Haghdoost, Ali Akbar; Momeni, Mohsen; Danaei, Mina	2018	32	Iran	F	Hematological	1
A case of radiation-induced bullous morphea/lichen sclerosus overlap in a breast cancer patient.	Morphea	3	Petersen, Erik; Yazdani, Laila; Hymes, Sharon R	2018	n/a	USA	F	Breast	1
Relapse of morphea during Nivolumab therapy for lung adenocarcinoma.	Morphea	3	Alegre-Sanchez, A; Fonda-Pascual, P;	2017	65	Spain	F	Lung	1

			Saceda-Corralo, D; de Las Heras-Alonso, E						
Characteristics and treatment of postirradiation morphea: A retrospective multicenter analysis.	Morphea	3	Fruchter, Renee; Kurtzman, Drew J B; Mazori, Daniel R; Wright, Natalie A; Patel, Mital; Vleugels, Ruth Ann; Femia, Alisa N	2017	n/a	USA	22F	Breast, parotid	22
Metastatic Squamous Cell Carcinoma in a Patient with Disabling Pansclerotic Morphea of Childhood.	Morphea	1	Ruiz-Matta, Juan M; Flores-Bozo, Luis R; Dominguez-Cherit, Judith	2017	23	Mexico	M	SCC	1
Radiotherapy-induced morphoea of the breast responding to photodynamic therapy.	Morphea	3	Papanikolaou, M; Tsianou, Z; Skellett, A M; Murphy, J; Millington, G W M	2018	n/a	UK	F	Breast	1
Underdiagnosed and disfiguring - Radiation-induced morphea following breast cancer treatment.	Morphea	3	Friedman, Or; Barnea, Yoav; Hafner, Ariela	2018	31-69	Israel	3F	Breast	3
Radiation-induced morphea-a rare but severe late effect of adjuvant breast irradiation : Case report and review of the literature.	Morphea	3	Partl, Richard; Regitnig, Peter; Tauber, Gerlinde; Potscher, Michaela; Bjelic-Radisic, Vesna; Kapp, Karin S	2018	72	Austria	F	Breast	1
Postirradiation morphea: unique presentation on the breast.	Morphea	3	Franco, Loren; Hausauer, Amelia K; Patel, Rishi R; Guth, Amber A; McLellan, Beth N	2018	62	USA	F	Breast	1
Localized morphea after breast implant for breast cancer: A case report.	Morphea	3	Moretti, A; Bianchi, F; Abbate, I V; Gherardi, G; Bonavita, M; Passoni, E; Nazzaro, G; Bramati, A; Dazzani, M C; Piva, S; Paterno, E;	2018	37	Italy	F	Breast	1

			Frungillo, N; Farina, G; La Verde, N						
Generalized morphea in the setting of pembrolizumab.	Morphea	3	Cheng, Michelle W; Hisaw, Lisa D; Bernet, Laura	2019	n/a	USA	M	Melanoma	1
Morphea and systemic sclerosis are associated with an increased risk for melanoma and nonmelanoma skin cancer.	Morphea	1	Boozalis, Emily; Shah, Ami A; Wigley, Fredrick; Kang, Sewon; Kwatra, Shawn G	2019	N/A	USA	81F, 8M	Melanoma, BCC, SCC	89
Postirradiation Morphea in Patients With Breast Cancer: Possible Association With Other Autoimmune Diseases.	Morphea	3	Diago, A; Llombart, B; Requena, C; Sanmartin, O; Guillen, C	2019	n/a	Spain	6F	Breast	6
Radiation-induced morphea: autoimmunity as a risk factor.	Morphea	3	Machan, A; Oumakhir, S; Khalidi, M; Hjira, N; Boui, M	2019	n/a	Morocco	F	Breast	1
Radiation-induced morphea: Association with autoimmune comorbidities, severity, and response to therapy.	Morphea	3	Mittal, Amit; Mittal, Vaishali; Panse, Gauri; Choi, Jennifer N; Kwong, Bernice Y; Leventhal, Jonathan S	2019	n/a	USA	23F, 2M	Breast	25
Generalised morphea induced by pembrolizumab.	Morphea	3	Herrscher, Hugo; Tomasic, Gorana; Castro Gordon, Alicia	2019	74	France	F	Melanoma	1
Nivolumab induced morphea.	Morphea	3	Dal Bello, Giacomo; Rosina, Paolo; Colato, Chiara; Girolomoni, Giampiero	2020	n/a	Italy	F	Melanoma	1
Localized scleroderma histologically characterized by liquefaction degeneration and upper dermis fibrosis: a possible association with chemotherapy.	Morphea	3	Toyama, S; Sato, S; Asano, Y	2020	58	Japan	M	Colon	1

Early morphea during treatment with ibrutinib in a patient with chronic lymphocytic leukemia.	Morphea	3	Ozbagcivan, Ozlem; Akarsu, Sevgi; Nazli, Ecem Canturk; Lebe, Banu; Fetil, Emel	2021	70	Turkey	M	hematological	1
Nivolumab-induced plaque morphea in a malign melanoma patient.	Morphea	3	Acar, Ayda; Oraloglu, Gokturk; Yaman, Banu; Karaarslan, Isil	2021	46	Turkey	F	Melanoma	1
Radiation-induced morphea and dystrophic calcinosis cutis of the breast.	Morphea	3	Perna, Danielle; Margheim, Ashlee; Schadt, Courtney R	2021	N/A	USA	F	Breast	1
Radiation-induced morphea - a rare, but not to be dismissed, adverse effect of radiotherapy.	Morphea	3	Gambichler, Thilo; Scheel, Christina H; Boms, Stefanie	2021	n/a	Germany	F	Breast	1
Generalized morphoea in the setting of combined immune checkpoint inhibitor therapy for metastatic melanoma: A case report.	Morphea	3	Langan, Ewan A; Budner, Kaja; Zillikens, Detlef; Terheyden, Patrick	2021	61	Germany	F	Melanoma	1
Nodular morphea keloidal type: A rare case with paradigmatic histopathology significantly accompanied by a flawless surgical scar.	Morphea	3	Castelli, Elena; Orlando, Elisabetta; Pardo, Nicola; Turdo, Alice; Pistone, Giuseppe; Bongiorno, Maria Rita	2021	40	Italy	F	Breast	1
Development of morphea during Nivolumab treatment.	Morphea	3	Fabregat-Pratdepadua, Maria; Boada, Aram; Manzano, Jose Luis; Bielsa, Isabel; Verdaguer-Faja, Julia; Quer Pi-Sunyer, Ariadna; Carrascosa, Jose-Manuel	2022	72	Spain	F	Melanoma	1
CAN SKIN BE A MARKER FOR INTERNAL MALIGNANCY? EVIDENCE FROM CLINICAL CASES.	Morphea	2	Lugovic-Mihic, Liborija; Kristo, Mirela; Spoljar, Sanja; Novak-Bilic,	2021	n/a	Croatia	F	Bladder	1

			Gaby; Beslic, Iva; Vucic, Majda; Situm, Mirna						
Incidence of Morphea following Adjuvant Irradiation of the Breast in 2,268 Patients.	Morphea	3	Partl, Richard; Regitnig, Peter; Lukasiak, Katarzyna; Winkler, Peter; Kapp, Karin Sigrid	2020	n/a	Austria	6F	Breast	6
Postradiation breast erythema, skin thickening, and peau d'orange.	Morphea	3	DeKraker, Corina; Gomez, Jose A; Arifin, Andrew; Perera, Francisco E	2022	68	Canada	F	Breast	1
Skin eruption involving bilateral breasts following radiation therapy for invasive ductal carcinoma of the left breast.	Morphea	3	Belzer, Annika; McNiff, Jennifer M; Leventhal, Jonathan S	2022	n/a	USA	F	Breast	1
Post-Irradiation Morphea of the Breast in a Patient with Subacute Cutaneous Lupus Erythematosus: Case Report and a Literature Review.	Morphea	3	Zahn, Carole Anouk; Feldmeyer, Laurence; Blum, Roland; Mainetti, Carlo	2022	68	Switzerland	F	Breast	1
Nivolumab-Induced Disseminated Morphea: A Previously Unreported Presentation of an Unusual Condition.	Morphea	3	Montero-Menarguez, Julia; Arroyo-Andres, Jorge; Fulgencio-Barbarin, Jon; Rodriguez-Peralto, Jose-Luis; Ortiz-Romero, Pablo Luis; Falkenhain-Lopez, Daniel	2022	53	Spain	F	Rectum	1
Autoimmune conditions and pancreatic cancer risk in older American adults.	Morphea	1	Yuan, Fangcheng; Pfeiffer, Ruth M; Julian-Serrano, Sachelly; Arjani, Simran; Barrett, Michael J; Koshiol, Jill;	2022	66-99	USA	N/A	Pancreas	166

			Stolzenberg-Solomon, Rachael Z						
Development of squamous cell carcinoma in generalized morphea	Morphea	1	Laux B.; Brauniger W.	1985	65	Germany	F	SCC	1
Phenotyping of cutaneous toxicities in patients with metastatic malignant melanoma treated with immune checkpoint blockade therapy at a UK tertiary care centre	Morphea	3	Ye W.; Kim M.; Fairfax B.P.; Coupe N.; Payne M.J.; Matin R.N.	2022	N/A	UK	N/A	Melanoma	1
Atypical scleroderma associated with multiple myeloma.	Morphea	1	Nakanishi, H; Takehara, K; Soma, Y; Ishibashi, Y	1989	53	Japan	M	Hematological	1
Histopathological improvement of scleroderma induced by paclitaxel in a patient with breast cancer.	Morphea	3	Ohashi, Atsuko; Minagawa, Akane; Ashida, Atsuko; Koga, Hiroshi; Uhara, Hisashi; Okuyama, Ryuhei	2015	47	Japan	F	Breast	1
Radiation-induced morphea of the breast five years post-treatment: A case report	Morphea	3	Lam E.; Yee C.; Drost L.; Wong G.; McKenzie E.; Chow E.; Vesprini D.	2019	64	Canada	F	Breast	1
[Scleroderma and cancer of the stomach].	Morphea	1	Roge, J; Delavierre, P; Durand, H; Besancon-Lajeunesse, L	1971	N/A	France	M	Stomach	1
[Radiation-induced morphea].	Morphea	3	Fischer, M; Bormann, G; Wohlrab, J; Marsch, W C	1999	N/A	Germany	F	Breast	1
Long-standing morphea and the risk of squamous cell carcinoma of the skin	Morphea	1	Johannes Heck 1, Joanna Olk 1, Hermann Kneitz 1, Henning Hamm 1, Matthias Goebeler 1	2020	31-67	Germany	2F, 3M	SCC	5

[Pick-Herxheimer's scleroderma with epitheliomatous degeneration]	Morphea	1	J DUVERNE, M PRUNIERAS, R MOUNIER, CHARMION	1958	67	France	M	SCC	1
Development of a squamous cell carcinoma in generalized morphea	Morphea	1	Laux B, Bräuninger W	1985	65	Germany	F	SCC	1
Morphea of the breast–an uncommon cause of breast erythema	Morphea	3	Clark CJ, Wechter D	2010	66,77	USA	2F	Breast	2
A case of postirradiation morphea	Morphea	3	Park S, Kang Y, Kim S, Park H, Lee U, Jang S.	2009	N/A	Korea	F	Breast	1
Irradiation-induced morphea: x-rays as triggers of autoimmunity	Morphea	3	Laetsch B, Hofer T, Lombriser N, Lautenschlager S.	2011	46-83	Switzerland	3F	Breast	3
Breast erythema and induration in a 77-year-old woman	Morphea	3	Woo DK, Reilly GD, Elenitsas R	2011	77	USA	F	Breast	1
Imaging surveillance of the breast in a patient diagnosed with scleroderma after breast-conserving surgry and radiotherapy	Morphea	3	Seale M, Koh W, Henderson M, Drummond R, Cawson J.	2008	55	Australia	F	Breast	1
A case of radiation-induced morphea	Morphea	3	Kim J, Chong S, Park H, Oh S, Lee J, Cho B	2005	N/A	N/A	F	Breast	1
Mixed connective tissue disease and radiation toxicity. A case report	Morphea	3	Mayr NA, Riggs CE, Jr, Saag KG, Wen BC, Pennington EC, Hussey DH.	1997	N/A	USA	F	Breast	1
Pseudosclerodermatous panniculitis after irradiation: an usual complication of megavoltage treatment of breast carcinoma	Morphea	3	Winkelmann RK, Grado GL, Quimby SR, Connolly SM.	1993	N/A	USA	F	Breast	4
Breast conservation therapy. Severe breast fibrosis after radioation therapy in patients with collagen vascular disease	Morphea	3	Robertson JM, Clarke DH, Pevzner MM, Matter RC	1991	N/A	USA	F	Breast	2

Atezolizumab-induced scleroderma: a rare complication	Morphea	3	Christon Grant,1<br><br>Varun Chalasani,2<br><br>Jeffrey M Uchin,3<br><br>Adam Dore1	2021	60	USA	F	Lung	1
Morphea following treatment with pembrolizumab for melanoma with metastatic lymph nodes: case report and review of literature	Morphea	3	Faraaz S Zafar 1, Patricia Richey 1, Uchenna Okereke 1, Mohammed Milhem 2, Roshan Abid 1, Jennifer G Powers	2021	31	USA	F	Melanoma	1
Scleroderma-like Syndrome in the<br>Setting of Pembrolizumab Therapy for<br>Non-Small Cell Lung Cancer: Diagnosis<br>and Dermatologic Management	Morphea	3	Davide Fattore Teresa Battista Mario De Lucia Maria Carmela Annunziata Gabriella Fabbrocini	2022	N/A	Italy	F	Lung	1
Skin toxicities compromise prolonged pemetrexed treatment	Morphea	3	Belen Eguia 1, Anne-Marie Ruppert, Julie Fillon, Armelle Lavolé, Valérie Gounant, Christelle Epaud, Bernard Milleron, Philippe Moguelet, Marie Wislez, Camille Frances, Jacques Cadranel	2011	N/A	France	N/A	Lung	1
Painful generalized erythematous patches: a severe and unusual cutaneous reaction to pemetrexed	Morphea	3	Magalys Vitiello, Paolo Romanelli, Francisco A Kerdel	2011	36	USA	F	Lung	1
Pemetrexed-induced skin sclerosis.	Morphea	3	C Merklen-Djafri, E Imbert, D Courouge-Dorcier, R Schott, J-P Méraud, C Muller, M Tebacher, G Springinsfeld, B Cribier, D Lipsker	2012	57	France	M	Lung	1

[Erythema and papulonecrotic, ulcerating and generalized dermatosis due to arteritis and chronic thrombarteritis]	Morphea	1	E FREY	1952	41	N/A	F	SCC	1
[Pemetrexed-induced scleroderma-like changes in the lower legs]	Morphea	3	C Corbaux 1, J Marie 2, J-P Meraud 2, S Lacroix 3, J-Y Delhoume 3, T Jouary 4, S Madoui 3	2015	66	France	M	Lung	2
Scleroderma-like lesions and Epstein-Barr virus associated B-cell lymphoma presenting with a huge splenomegaly	Eosinophilic Fasciitis	1	Sakai C.; Takagi T.; Wakatsuki S.; Matsuzaki O.	1999	66 (1st cancer) 74 (2nd cancer)	N/A	F	Multiple	1
Skin puckering and edema during durvalumab therapy	Eosinophilic Fasciitis	3	Olamiju B.; Odell I.; Panse G.; Eder J.P.; Leventhal J.S.	2021	N/A	USA	F	Solid	1
Rheumatic manifestations among cancer patients treated with immune checkpoint inhibitors.	Eosinophilic Fasciitis	3	Lidar, Merav; Giat, Eitan; Garelick, Daniela; Horowitz, Yuval; Amital, Howard; SteinbergSilman, Yael; Schachter, Jacob; ShapiraFrommer, Ronnie; Markel, Gal	2018	N/A	Israel	F	Solid	1
Response of eosinophilic fasciitis associated with Waldenstrom macroglobulinemia to rituximab.	Eosinophilic Fasciitis	2	Kromer, Christian; Matzke, Silke S; Bleckmann, Anlen; Overbeck, Tobias; Lippert, Undine; Schon, Michael P; Mossner, Rotraut	2021	66	Germany	M	Hematological	1
Postradiation scleredema adultorum and diffuse eosinophilic fasciitis in the same patient.	Eosinophilic Fasciitis	3	Mylo, E; Golfinopoulou, S; Skarmea, A; Katsikas, G; Skrepetou, K; kos, G; Tsigka, A; Skoutelis, A	2011	60	Greece	F	Solid	1
Patients with eosinophilic fasciitis should have a bone marrow examition to identify myelodysplasia [9]	Eosinophilic Fasciitis	2	BritoBabapulle F.	1997	84	UK	M	Hematological	1
Paraneoplastic scleroderma-like tissue reactions in the setting of	Eosinophilic Fasciitis	2	Magro, Cynthia M; Iwenofu, Hans; Nuovo, Gerard J	2013	68, 85	USA	F, M	Hematological	2

an underlying plasma cell dyscrasia: a report of 10 cases.									
Paraneoplastic fasciitis - Panniculitis syndrome: A neurological point of view	Eosinophilic Fasciitis	2	Dikar P.; Hoke A.	2009	49	USA	F	Solid	1
Paraneoplastic eosinophilic fasciitis: a case report.	Eosinophilic Fasciitis	2	Jacob, Sharon E; Lodha, Rashmi; Cohen, Jothan J; Romanelli, Paolo; Kirsner, Robert S	2003	40	USA	F	Hematological	1
Paraneoplastic eosinophilic fasciitis with generalized morphea and vitiligo in a patient working with organic solvents.	Eosinophilic Fasciitis	1	Hami, Yuka; Ohtsuka, Mikio; Yamamoto, Toshiyuki	2016	64	Japan	M	Hematological	1
Nivolumab-induced multiple morphea associated with eosinophilic fasciitis.	Eosinophilic Fasciitis	3	Lacombe, Marouschka; Bedane, Christophe; Delaumenie, Stephanie; Berrd, Philippe	2021	50	France	M	Solid	1
Nivolumab-induced eosinophilic fasciitis: a case report.	Eosinophilic Fasciitis	3	Ollier, tasha; Tournier, Emilie; Meyer, Nicolas; Sibaud, Vincent; PagesLaurent, Cecile; Cougoul, Pierre; BeyneRauzy, Odile; Comont, Thibault	2020	35 at melanoma 61 at papillary 64 at melanoma reccurrence	France	M	Multiple	1
Lymph-node-based malignant lymphoma and reactive lymphadenopathy in eosinophilic fasciitis	Eosinophilic Fasciitis	2	schitz J.E.; Misselevich I.; Rosner I.; Yeshurun D.; Weiner P.; Amar M.; Amato L.; Ciompi M.L.; Boss J.H.	1999	72, N/A	N/A	F, F	Solid, Hematological	2
Long-term outcome of eosinophilic fasciitis: A cross-sectional evaluation of 35 patients	Eosinophilic Fasciitis	2	Mertens J.S.; Thurlings R.M.; Kievit W.; Seyger M.M.B.; Radstake T.R.D.; de Jong E.M.G.J.	2017	N/A	Netherlands	N/A	Solid	2
Immune Checkpoint Inhibitor-Associated Scleroderma-Like Syndrome: A Report of a Pembrolizumab-Induced "Eosinophilic Fasciitis-Like" Case and a Review of the Literature.	Eosinophilic Fasciitis	3	Salamaliki, Christi; Solomou, Ele E; Liossis, StamatisNick C	2020	80	Greece	M	Solid	1

Forearm compartment syndrome as a result of eosinophilic fasciitis: case report.	Eosinophilic Fasciitis	3	Alolabi, Bashar; Lesieur, Martin; Smilovici, Brian; Koo, Kevin; El Bahtimi, Reem; Jenkinson, Richard J	2015	N/A	CaN/Ada	F	Hematological	1
Familial eosinophilic fasciitis and breast cancer.	Eosinophilic Fasciitis	2,3	Watts, R A; Merry, P	1994	56	England	2F	Solid	2
Eosinophilic fasciitis/generalized morphea overlap successfully treated with azathioprine	Eosinophilic Fasciitis	1	AlonsoCastro L.; de las Heras E.; Moreno C.; FletaAsin B.; MunozZato E.; Carrillo R.; Jaen P.	2014	N/A	Spain	M	Solid	1
Eosinophilic fasciitis: clinical spectrum and therapeutic response in 52 cases.	Eosinophilic Fasciitis	2	Lakhanpal, S; Ginsburg, W W; Michet, C J; Doyle, J A; Moore, S B	1988	N/A	USA	F, N/A, N/A, N/A	Solid, hematological, hematological, hematological	4
Eosinophilic fasciitis, myositis and arthritis as early manifestations of peripheral Tcell lymphoma [2]	Eosinophilic Fasciitis	2	Eklund K.; Anttila P.; LeirisaloRepo M.	2003	56	Finland	F	Hematological	1
Eosinophilic fasciitis with severe joint contracture in a patient with bladder cancer and B-cell lymphoma.	Eosinophilic Fasciitis	2	Hiraiwa, Tomoko; Mori, Tatsuhiko; Ohashi, Takenobu; Hami, Yuka; Yamamoto, Toshiyuki	2016	81 and lymphoma dx < 79 at bladder cancer dx	Japan	M	Multiple	1
Eosinophilic fasciitis with myelodysplasia responsive to treatment with cyclosporin [13]	Eosinophilic Fasciitis	2	Fleming C.J.; Clarke P.; Kemmett D.	1997	76	Scotland	F	Hematological	1
Eosinophilic Fasciitis with Hypereosinophilia as the Initial Clinical Manifestation of Peripheral T-Cell Lymphoma, Not Otherwise Specified.	Eosinophilic Fasciitis	2	Okuyama, Shuhei; Satomi, Hidetoshi; Ishikawa, Ryuto; Shishido, Tsutomu; Sato, Keijiro; Ueki, Toshimitsu; Sumi, Masahiko; Kobayashi, Hikaru	2022	58	Japan	M	Hematological	1
Eosinophilic Fasciitis with Concurrent Necrobiotic Granulomatous Dermatitis	Eosinophilic Fasciitis	3	Haroon, Adeeb; Tadros, Joseph; Smith, Emily H	2022	N/A	USA	M	Solid	1

Related to Checkpoint Inhibition Therapy.									
Eosinophilic Fasciitis With a Malignant Outcome.	Eosinophilic Fasciitis	1	Ansari, Salman; Iftikhar, Umair; Jamil, Asma; Ansari, Aamir; Iftikhar, Syed	2021	77	USA	F	Non-melanoma skin	1
Eosinophilic Fasciitis Unmasking a Lung Cancer.	Eosinophilic Fasciitis	2	Vandamme, Emilie; Haiut, Philippe	2022	60	Belgium	M	Solid	1
Eosinophilic fasciitis preceding relapse of peripheral T-cell lymphoma.	Eosinophilic Fasciitis	2	Kim, H; Kim, M O; Ahn, M J; Lee, Y Y; Jung, T J; Choi, I Y; Kim, I S; Park, C K	2000	65 at relapse	Korea	M	Hematological	1
Eosinophilic fasciitis induced by nivolumab therapy managed without treatment interruption or systemic immunosuppression.	Eosinophilic Fasciitis	3	Bui, AiTram N; Nelson, Caroline A; Lian, Christine G; Cales, Alvaro Laga; LeBoeuf, Nicole R	2020	N/A	USA	M	Solid	1
Eosinophilic fasciitis in association with nivolumab: The importance of eosinophilia.	Eosinophilic Fasciitis	3	PabonCartage, Gabriela; Lopez, Andrea; Watts, Erika; Alonso, Norma	2020	N/A	Puerto Rico	F	Non-melanoma skin	1
Eosinophilic Fasciitis Following Checkpoint Inhibitor Therapy: Four Cases and a Review of Literature	Eosinophilic Fasciitis	3	Chan K.K.; Magro C.; Shoushtari A.; Rudin C.; Rotemberg V.; Rossi A.; Lezcano C.; Carrino J.; Ferndez D.; Postow M.A.; Apollo A.; Lacouture M.E.; Bass A.R.	2020	N/A, 69, 43, 70	N/A	M, F, M, M	Solid	4
Eosinophilic Fasciitis following Checkpoint Inhibitor Therapy with Pembrolizumab.	Eosinophilic Fasciitis	3	Zampeli, Evangelia; Zervas, Eleftherios	2021	60	Greece	M	Solid	1
Eosinophilic fasciitis complicated by Hodgkin's disease.	Eosinophilic Fasciitis	1	Michaels, R M	1982	56	USA	F	Hematological	1
Eosinophilic fasciitis associated with low-grade T-cell lymphoma [11]	Eosinophilic Fasciitis	2	Masuoka H.; Kikuchi K.; Takahashi S.; Kakinuma T.; Hayashi N.; Furue M.	1998	51	Japan	F	Hematological	1
Eosinophilic fasciitis associated with hypereosinophilia, abnormal bone-marrow karyotype and inversion of chromosome 5.	Eosinophilic Fasciitis	2	Ferguson, J S; Bosworth, J; Min, T; Mercieca, J; Holden, C A	2014	N/A	UK	M	Hematological	1

Eosinophilic fasciitis associated with autoimmune thyroid disease and myelodysplasia treated with pulsed methylprednisolone and antihistamines.	Eosinophilic Fasciitis	2	Farrell, A M; Ross, J S; Bunker, C B	1999	N/A	UK	M	Hematological	1
Eosinophilic fasciitis as a paraneoplastic syndrome, a case report and review of the literature	Eosinophilic Fasciitis	1	Haddad H.; Sundaram S.; Magro C.; Gergis U.	2014	70 at AML dx	N/A	M	Multiple	1
Eosinophilic fasciitis as a paraneoplastic syndrome in melanoma	Eosinophilic Fasciitis	2	Rea A.; Anderson A.; Moshiri A.; Paulson K.; Thompson J.A.; Kalus A.	2021	72	USA	F	Solid	1
Eosinophilic fasciitis as a paraneoplastic phenomenon associated with metastatic colorectal carcinoma.	Eosinophilic Fasciitis	2	Philpott, Hamish; Hissaria, Pravin; Warrren, Lachlan; Singhal, Nimit; Brown, Michael; Proudman, Susan; Cleland, Les; Gillis, David	2008	68	Australia	M	Solid	1
Eosinophilic fasciitis as a manifestation of a cutaneous T-cell lymphoma not otherwise specified.	Eosinophilic Fasciitis	2	CastellanosGonzalez, Maria; Velasco Rodriguez, Diego; Blanco Echevarria, Agustin; Postigo, Concha; Ortiz Romero, Pablo; Diaz, Rosa Ayala; RodriguezPeralto, Jose Luis	2013	76	Spain	M	Hematological	1
Eosinophilic Fasciitis and Smoldering Multiple Myeloma: An Exceptional Association in Young Adults	Eosinophilic Fasciitis	2	Jabbouri, Rajaa; Bouani, Nouama; Aniq Filali, Rita; Aasfara, Jehanne	2022	24	Morocco	M	Hematological	1
Eosinophilic fasciitis and non-Hodgkin lymphoma.	Eosinophilic Fasciitis	2	Junca, J; Cuxart, A; Tural, C; Ojanguren, I; Flores, A	1994	71	Spain	F	Hematological	1
Eosinophilic fasciitis and lichen sclerosus in a patient treated with nivolumab.	Eosinophilic Fasciitis	3	AndresLenci, JuanJose; BurilloMartinez, Sara; AragonMiguel, Raquel; CallejaAlgarra, Alba; RodriguezPeralto, JoseLuis; OrtizRomero,	2018	63	Spain	M	Solid	1

			PabloLuis; GargalloMoneva, Vanessa						
Eosinophilic Fasciitis and Acute Encephalopathy Toxicity from Pembrolizumab Treatment of a Patient with Metastatic Melanoma.	Eosinophilic Fasciitis	3	Khoja, Leila; Maurice, Catherine; Chappell, MaryAnne; MacMillan, Leslie; AlHabeeb, Ayman S; AlFaraidy, da; Butler, Marcus O; Rogalla, Patrik; Mason, Warren; Joshua, Anthony M; Hogg, David	2016	51	CaN/Ada	F	Solid	1
Drug-Associated Eosinophilic Fasciitis: A Case of Eosinophilic Fasciitis Secondary to Cemiplimab Therapy.	Eosinophilic Fasciitis	3	Boppa, Sri Harsha; Dulla, geswara Rao; Beutler, Bryce D; Gullapalli, geshwara; Kaur, Ratinder	2021	N/A	N/A	F	Non-melanoma skin	1
Diffuse fasciitis with eosinophilia developing after local irradiation for breast cancer.	Eosinophilic Fasciitis	3	Sherber, Noelle S; Wigley, Fredrick M; Paget, Stephen A	2009	66	USA	F	Solid	1
Concurrent eosinophilic fasciitis and cutaneous T-cell lymphoma. Eosinophilic fasciitis as a paraneoplastic syndrome of T-cell malignant neoplasms.	Eosinophilic Fasciitis	2	Chan, L S; Hanson, C A; Cooper, K D	1991	73	USA	M	Hematological	1
Complete reversibility of pembrolizumab-induced eosinophilic fasciitis without corticosteroids: A case report.	Eosinophilic Fasciitis	3	Bourcier, Liane; StHilaire, Eve; LeBlanc, Martin; Picard, Leo	2021	25	CaN/Ada	M	Solid	1
Checkpoint inhibitor-induced eosinophilic fasciitis following high eosinophilia associated with complete response.	Eosinophilic Fasciitis	3	Toussaint, Frederic; Hammon, Matthias; Erdmann, Michael; Moreira, Alvaro; Kirchberger, Michael C; Schuler, Gerold; Schett, Georg; Heinzerling, Lucie	2019	N/A	N/A	F	Solid	1
Cancer associated fasciitis panniculitis	Eosinophilic Fasciitis	2	schitz J.E.; Yeshurun D.; Zuckerman E.; Rosenbaum M.; Misselevitch I.; Shajrawi I.; Boss J.H.	1994	72, 80, 59	Israel	F, M, F	Solid, solid, hematological	3

Bone marrow transplantation in a patient with myelodysplasia associated with diffuse eosinophilic fasciitis.	Eosinophilic Fasciitis	2	Tallman, M S; McGuffin, R W; Higano, C S; Starkebaum, G; Collins, S J; Johnston, H; Singer, J W; Perry, D J; Kuth, A	1987	35	USA	M	Hematological	1
Baseline characteristics and long-term outcomes of eosinophilic fasciitis in 89 patients seen at a single center over 20 years	Eosinophilic Fasciitis	2	Mango R.L.; Bugdayli K.; Crowson C.S.; Drage L.A.; Wetter D.A.; Lehman J.S.; Peters M.S.; Davis M.D.; Chowdhary V.R.	2020	N/A	USA	N/A	Hematological x6, solid x5, non-melanoma skin x1	12
An Original Case of an Association of Eosinophilic Fasciitis with Cholangitis Induced by Nivolumab.	Eosinophilic Fasciitis	3	Le Tallec, Erwan; Ricordel, Charles; Triquet, Louise; Deniel, Arud; Marcorelles, Pascale; Le, Herve; Jego, Patrick; Belhomme, Nicolas	2019	N/A	France	F	Solid	1
[Eosinophilic fasciitis and metastatic choroidal melanoma: a paraneoplastic syndrome?].	Eosinophilic Fasciitis	3	VeyssierBelot, C; Zuech, P; LumbrosoLe Rouic, L; Recati, G; Dendale, R	2008	67 at metastases	France	F	Solid	1
[Eosinophilic fasciitis and Hodgkin's disease].	Eosinophilic Fasciitis	1	Rodat, O; Harousseau, J L; Reyud, C; Milpied, N; Stalder, J F; Chupin, M	1982	73	France	F	Hematological	1
Eosinophilic fasciitis with multiple myeloma: a new haematological association	Eosinophilic Fasciitis	2	Khan, D; Verity, A; Grossman, J M	2002	48	USA	F	Hematological	1
[Chronic sclerodermiform syndrome disclosing subcutaneous T-cell lymphoma]	Eosinophilic Fasciitis	2	Cocciale M.P.; Plet F.; Reichert S.; Modiano P.; Barbaud A.; Weber M.; Schmutz J.L.	1997	55	France	F	Hematological	1
