# Supplementary material for: Morphea, Eosinophilic Fasciitis and Cancer: A Scoping Review
Source: Cancers (Basel). 2023 Sep 7;15(18):4450. doi: 10.3390/cancers15184450 (PMC10526289; doi:10.3390/cancers15184450)
Supplement: Supplementary file 1 [file cancers-15-04450-s001.zip › Supplementary_Material_Table_Ss_morphea_cancers.pdf]

Supplementary Material Table S2. Multimodal cancer treatment.

Article title	Authors	Cancer Site	Age at cancer diagnosis	Time to LS onset from cancer treatment (months)	Number of patients	Radiotherapy	Chemotherapy	Surgery	Immunotherapy	Main treatment suspected to have induced LS
Sclerodermiform aspect of arm lymphoedema after treatment with docetaxel for breast cancer.	Vignes et al	Breast	58	Few months	1	Yes	Yes	Yes	No	Docetaxel
		Breast	54		1	Yes	Yes	Yes	No	Docetaxel
		Breast	56		1	Yes	Yes	Yes	No	Docetaxel
		Breast	44		1	No	Yes	Yes	No	Docetaxel
		Breast	44		1	Yes	Yes	Yes	No	Docetaxel
		Breast	48		1	Yes	Yes	Yes	No	Docetaxel
		Breast	65		1	Yes	Yes	Yes	No	Docetaxel
		Breast	53		1	No	Yes	Yes	No	Docetaxel
		Breast	56		1	No	Yes	Yes	No	Docetaxel
		Breast	55		1	Yes	Yes	Yes	No	Docetaxel
		Breast	60		1	Yes	Yes	Yes	No	Docetaxel
Pemetrexed-induced scleroderma-like conditions in the lower legs of a patient with non-small cell lung carcinoma.	Ishikawa et al	Lung	63	24	1	Yes	Yes	Yes	No	Pemetrexed
Disseminated morphea in small cell lung cancer [5]	Benekli et al	Lung	34	12	1	Yes	Yes	No	No	Radiation
Postirradiation morphea in breast cancer	Martin et al	Breast	56	24	1	Yes	Yes	Yes	No	Radiation
Postirradiation morphea and subcutaneous	Reddy et al	Breast	75	4	1	Yes	Yes	No	No	Radiation

polyarteritis nodosa: Case report and literature review										
Post-irradiation morphea of the breast: does this pose an issue for reconstruction?	Rafique et al	Breast	52	3.5	1	Yes	Yes	No	No	Radiation
Post radiotherapy deep morphea. A case report	Corball et al	Breast	n/a	96	1	Yes	Yes	Yes	No	Radiation
Postirradiation morphea: A case report with a review of the literature and summary of the clinicopathologic differential diagnosis	Morganroth et al	Breast	45	72	1	Yes	Yes	Yes	No	Radiation
Circumscribed scleroderma induced by postlumpectomy radiation therapy.	Trattner et al	Breast	55	Few months	1	Yes	No	Yes	No	Radiation
Widespread morphoea following radiotherapy for carcinoma of the breast.	Ardern-Jones et al	Breast	60	Few years	1	Yes	Yes	Yes	No	Radiation
Post-Irradiation Morphea in Breast Cancer: An Uncommon Differential Diagnosis to Keep in Mind.	Afonso-Afonso et al	Breast	51	11	1	Yes	Yes	No	No	Radiation
Underdiagnosed and disfiguring - Radiation-induced morphea following breast cancer treatment.	Friedman et al	Breast	42	48	1	Yes	Yes	Yes	No	Radiation

Radiation-induced morphea: autoimmunity as a risk factor.	Machan et al	Breast	53	48	1	Yes	Yes	Yes	No	Radiation
Generalized morphoea in the setting of combined immune checkpoint inhibitor therapy for metastatic melanoma: A case report.	Langan and al	Melanoma	61	10	1	Yes	Yes	Yes	Nivolumab + pembrolizumab + ipilimumab	Combined immunotherapy
Postradiation breast erythema, skin thickening, and peau d'orange.	DeKraker et al	Breast	68	12	1	Yes	Yes	Yes	No	Radiation
Docetaxel (Taxotere) associated scleroderma-like changes of the lower extremities. A report of three cases.	Battafarano et al	Breast	63	Few months	1	Yes	Yes	No	No	Docetaxel
Localized scleroderma in breast cancer patients treated with supervoltage external beam radiation: radiation port scleroderma.	Davis et al	Breast	52	Less than 12 months	6	Yes	Yes	No	No	Radiation
Radiation-induced morphea of the breast: a case report.	Cheah et al	Breast	57	9	1	Yes	Yes	No	No	Radiation
Imaging surveillance of the breast in a patient diagnosed with scleroderma after breast-conserving surgry and radiotherapy	Seale et al	Breast	55	6	1	Yes	Yes	Yes	No	Radiation + Surgery

Post-irradiation morphoea.	Colver et al	Breast	55	36	1	Yes	Yes	Yes	No	Radiotherapy possibly aggraved by chemotherapy
Drug-induced localised scleroderma	Maya et al	Breast	70	1	1	No	Yes	Yes	Yes	All drugs
Vitiligo-like depigmentation and morpheas after specific intralymphatic immunotherapy for malignant melanoma.	Lacour et al	Melanoma	66	24	1	No	Yes	Yes	Yes	Immunotherapy
Breast cancer and scleroderma.	Scope et al	Breast	43	96	1	Yes	Yes	Yes	No	NA
[Post-irradiation morphea in brest cancer: a case report].	Llenas et al	Breast	NA	48	1	Yes	Yes	No	No	Radiation
Morphea following radiation therapy in a patient with breast cancer.	Garcia-Arpa et al	Breast	72	NA	1	Yes	Yes	No	No	Radiation
Radiation-induced morphea-a rare but severe late effect of adjuvant breast irradiation : Case report and review of the literature.	Partl et al	Breast	72	72	1	Yes	No	No	Yes	Radiation + Immunotherapy
Localized morphea after breast implant for breast cancer: A case report.	Moretti et al	Breast	37	5	1	No	Yes	Yes	No	Surgery + Chemotherapy
Localized morphea after silicone-gel-filled breast implant	Granel et al	Breast	53	12	1	No	Yes	Yes	No	Surgery + Chemotherapy
