# Supplementary material for: Morphea, Eosinophilic Fasciitis and Cancer: A Scoping Review
Source: Cancers (Basel). 2023 Sep 7;15(18):4450. doi: 10.3390/cancers15184450 (PMC10526289; doi:10.3390/cancers15184450)
Supplement: Supplementary file 1 [file cancers-15-04450-s001.zip › Supplementary_methods morphea cancers_Revised_MJC.pdf]

Supplementary Methods

Inclusion criteria:

- Adult and pediatric population diagnosed with morphea and cancer
- All dates
- Original articles
- All languages
- Human studies

Exclusion criteria:

- Articles' titles and/or abstracts that do not report on LS or cancer
- Articles that report on scleroderma-like conditions
- Review articles
- Conference abstract articles

The following conditions were excluded: systemic sclerosis (SSc) and sclerodermoid conditions related to other disease (amyloidosis, graft versus host disease (GVHD), carcinoid, lsc&a if no concurrent LS, borreliosis), non-malignancy like benign tumors (myelofibrosis x2 cases, neurofibroma x 1 case, lymphoid hyperplasia x1, carcinoid x2, thymoma x1, ankylosing spondylitis x1, cavernomas x1, uveitis x1, MS x2, fibrous hamartoma x1).
